# Identification and characterization of CHCHD1, AURKAIP1, and CRIF1 as new members of the mammalian mitochondrial ribosome

**DOI:** 10.3389/fphys.2013.00183

**Published:** 2013-07-30

**Authors:** Emine C. Koc, Huseyin Cimen, Beril Kumcuoglu, Nadiah Abu, Gurler Akpinar, Md. Emdadul Haque, Linda L. Spremulli, Hasan Koc

**Affiliations:** ^1^Department of Biochemistry and Microbiology, Joan C. Edwards School of Medicine, Marshall UniversityHuntington, WV, USA; ^2^Department of Biochemistry and Molecular Biology, Pennsylvania State UniversityUniversity Park, PA, USA; ^3^Department of Chemistry, University of North CarolinaChapel Hill, NC, USA; ^4^Department of Pharmaceutical Science and Research, School of Pharmacy, Marshall UniversityHuntington, WV, USA

**Keywords:** mitochondrial translation, MRPS37 (CHCHD1), MRPS38 (AURKAIP1), MRPS39 (PTCD3), MRPL58 (ICT1), MRPL59 (CRIF1), 55S, mitochondrial ribosomal proteins

## Abstract

Defects in mitochondrial ribosomal proteins (MRPs) cause various diseases in humans. Because of the essential role of MRPs in synthesizing the essential subunits of oxidative phosphorylation (OXPHOS) complexes, identifying all of the protein components involved in the mitochondrial translational machinery is critical. Initially, we identified 79 MRPs; however, identifying MRPs with no clear homologs in bacteria and yeast mitochondria was challenging, due to limited availability of expressed sequence tags (ESTs) in the databases available at that time. With the improvement in genome sequencing and increased sensitivity of mass spectrometry (MS)-based technologies, we have established four previously known proteins as MRPs and have confirmed the identification of ICT1 (MRP58) as a ribosomal protein. The newly identified MRPs are MRPS37 (Coiled-coil-helix-coiled-coil-helix domain containing protein 1-CHCHD1), MRPS38 (Aurora kinase A interacting protein1, AURKAIP1), MRPS39 (Pentatricopeptide repeat-containing protein 3, PTCD3), in the small subunit and MRPL59 (CR-6 interacting factor 1, CRIF1) in the large subunit. Furthermore, we have demonstrated the essential roles of CHCHD1, AURKAIP1, and CRIF1in mitochondrial protein synthesis by siRNA knock-down studies, which had significant effects on the expression of mitochondrially encoded proteins.

## Introduction

Mammalian mitochondria use oxidative phosphorylation (OXPHOS) to provide more than 90% of the ATP used for energy by cells. ATP production by this process depends on electron transport chain complexes and ATP synthase, components of which are encoded by both the nuclear and mitochondrial genomes. In mammals, mitochondrial DNA encodes the information for only 13 essential proteins required for OXPHOS, in addition to 22 tRNAs and 2 rRNAs. The 13 proteins encoded by the mitochondrial genome are synthesized on 55S ribosomes using the specialized translational system within the organelle.

Mammalian mitochondrial ribosomes (55S) consist of small (28S) and large (39S) subunits (O'Brien et al., 1993; Pel and Grivell, 1994). The 55S ribosome is composed of ~80 mitochondrial ribosomal proteins (MRPs), and all of these proteins are encoded by nuclear genes and imported into mitochondria, where they are assembled into the ribosome with mitochondrially transcribed rRNAs (De Vries and Van Der Koogh-Schuuring, 1973; Pietromonaco et al., 1991; Koc et al., 2001a,b). About a decade ago, we and others identified almost all of the proteins present in these ribosomes using various proteomics techniques. This initial analysis indicated that the small subunit of the ribosome contains 29 proteins, whereas the large subunit has about 50 proteins (Goldschmidt-Reisin et al., 1998; Graack et al., 1999; Koc et al., 1999, 2001a,b; Suzuki et al., 2001). About half of the proteins in mammalian mitochondrial ribosomes have homologs in bacterial and yeast mitochondrial ribosomes and play a role either in the assembly and structure of ribosomes or in the initiation, elongation, or termination phases of mitochondrial translation (Smits et al., 2007; Christian and Spremulli, 2011; Koc and Koc, 2012). The functions and ribosomal locations of the mitochondrial-specific proteins are not known; however, they may replace some of the functions of bacterial ribosomal proteins that are not present in the mitochondrial ribosome. They may also provide additional function(s) critical for protein synthesis or its regulation in mammalian mitochondria (Koc et al., 2001a,b). Although it is not possible to determine the exact locations of these mitochondrial-specific proteins without x-ray structural information, cryo-EM reconstruction studies indicate that they are distributed on the exterior surface of the ribosome (Sharma et al., 2003; Agrawal et al., 2011). It is clear that some of the mitochondrial-specific ribosomal proteins are located in functionally important regions of the ribosome, particularly at the mRNA entrance gate in the small subunit and at the polypeptide exit tunnel and central protuberance region of the large subunit, creating specific structures on the mitochondrial ribosome (Sharma et al., 2003; Agrawal et al., 2011).

In many instances, mitochondrial-specific ribosomal proteins were initially identified based on distinct biological functions that did not appear to be related to mitochondrial translation. For example, mitochondrial-specific MRPs such as MRPS29 (DAP3-death associated protein 3) and MRPS30 (PDCD9-programmed cell death protein 9) were originally identified on the basis of their involvement in apoptosis. MRPL37 and MRPL41 were also reported to be involved in apoptosis (Carim et al., 1999; Koc et al., 2001a,b; Levshenkova et al., 2004; Chintharlapalli et al., 2005; Yoo et al., 2005). Human mutations in MRPs have been reported and can have a variety of consequences (Galmiche et al., 2011; Smits et al., 2011). Major mutations in MRPs can lead to functional changes in mitochondrial translation and can be lethal (Miller et al., 2004; Jacobs and Turnbull, 2005; Saada et al., 2007; Galmiche et al., 2011; Rotig, 2011; Smits et al., 2011). Aberrantly expressed MRPs are also observed in many different tumors, including in breast cancer, gliomas, squamous cell carcinoma, and osteosarcoma (Bonnefoy et al., 2001; Koc et al., 2001b; Mariani et al., 2001; Miller et al., 2004; Lyng et al., 2006). Therefore, a complete list of mitochondrial ribosomal proteins will be fundamental to our understanding of the mitochondrial translational machinery and its contribution to mitochondrial ATP production in health and disease.

In the present study, we re-evaluated protein components of the mammalian mitochondrial ribosome using mass spectrometry (MS)-based proteomics and have established that five previously known mitochondrial proteins are components of the mitochondrial ribosome. These proteins are coiled-coil-helix-coiled-coil-helix domain containing protein 1 (CHCHD1), aurora kinase A interacting protein 1 (AURKAIP1), pentatricopeptide repeat-containing protein 3 (PTCD3), immature colon carcinoma transcript 1 protein (ICT1) and CR-6 interacting factor 1 [CRIF1, also known as growth arrest and DNA-damage-inducible proteins-interacting protein 1 (Gadd45GIP1)]. Three newly established mitochondrial ribosomal proteins (CHCHD1, AURKAIP1, and PTCD3) were assigned to the small subunit of the 55S ribosome, while ICT1 and CRIF1 were assigned to the large subunit. We further confirmed the specific roles for CHCHD1, AURKAIP1, and CRIF1 in mitochondrial translation by siRNA knock-down studies in human cell lines.

## Materials and methods

### Preparation of bovine mitochondrial ribosomal subunits

Mitochondrial ribosomes from bovine liver were prepared using a previously described method at high and low ionic strengths and at several different detergent concentrations (Matthews et al., 1982; Koc et al., 2001a,b; Spremulli, 2007). The high ionic strength and detergent concentrations used the standard conditions of 300 mM KCl and 1.6% Triton-X100 (Yang et al., 2010). For the preparation of mitochondrial ribosomes at low salt and detergent conditions, mitochondrial lysates were prepared in a buffer containing 50 mM Tris-HCl, pH 7.6, 40 mM KCl, 20 mM MgCl_2_, 6 mM β-mercaptoethanol, 0.2% Triton X-100, and 1 mM phenylmethylsulfonyl fluoride (PMSF). The mitochondrial lysate (100 mg/mL) was layered onto a 34% sucrose cushion (40 mM KCl, 20 mM MgCl_2_, 50 mM Tris-HCl, pH 7.6, 6 mM β-mercaptoethanol and 34% (w/w) sucrose). Samples were centrifuged at 35,000 rpm for 16 h at 4°C in a Beckman Type-50.2 rotor. The pellet was collected as crude mitochondrial ribosomes and resuspended in a buffer prepared with 40 mM KCl, 20 mM MgCl_2_, 20 mM Tris-HCl pH 7.6, 1 mM dithiothreitol (DTT), and protease inhibitor cocktail from Sigma-Aldrich (containing 1.04 mM AEBSF, 0.8 μ M Aprotinin, 40 μ M Bestatin, 14 μ M E-64, 20 μ M Leupeptin, and 15 μ M Pepstatin A as the final concentrations). Samples were then subjected to centrifugation through a 10–30% linear sucrose gradient in buffer containing 40 mM KCl, 20 mM MgCl_2_, 50 mM Tris-HCl pH 7.6, and 1 mM DTT as described (Matthews et al., 1982; Koc et al., 2001a,b; Spremulli, 2007). Fractions containing 55S ribosomes were combined, and the ribosomes were collected by centrifugation at 40,000 rpm for 16 h. The concentration of Mg^2+^ in the preparations was reduced to 2 mM by dialysis in order to dissociate ribosomes into 28S and 39S subunits, and the samples were separated again on a linear 10–30% sucrose gradient containing 2 mM Mg^2+^ as described (Matthews et al., 1982; Koc et al., 2001a,b; Spremulli, 2007). Highly purified 28S and 39S subunits were collected by centrifugation at 40,000 rpm for 16 h.

### Identification of mitochondrial ribosomal proteins by mass spectrometry

To identify the proteins of mammalian mitochondrial ribosomes, approximately 0.5 A_260_ units of purified ribosomal 28S and 39S subunits and 55S samples were separated on SDS-PAGE, and corresponding protein bands were excised into at least thirty equal gel pieces. The pieces were processed by performing in-gel tryptic digestion using methods previously established in our laboratory (Miller et al., 2008, 2009; Soung et al., 2009). Tryptic digests were analyzed by capillary liquid chromatography-nanoelectrospray ionization-tandem mass spectrometry (capLC-MS/MS). Extracted tryptic peptides (3–5 μ L) were injected into a peptide trap (Michrom peptide CapTrap, C8 like resin, 0.3 × 1 mm, 5 μm) over a 3 min interval at 10 μ L/min for online desalting and concentration. With the use of a six-port switching valve, the peptide trap was then placed in-line with the analytical column, a PicoFrit column (0.075 × 150 mm) packed in-house with Wide Bore C18 reverse phase resin (Supelco Co., 5 μm, 300 Å). Tandem MS spectra of tryptic peptides were obtained by collision-induced dissociation (CID) in an LTQ linear ion trap mass spectrometer system (ThermoFinnigan) including a Surveyor HPLC pump and a Surveyor Micro AS autosampler.

MS/MS spectra were processed by Xcalibur 2.0 and searched against the nonredundant NCBI protein database and the Swiss-Prot and UnitProtKB databases using the Mascot server. Additionally, the search was repeated using a bovine protein sequence database generated in-house. The protein information obtained from the database searches and the scores of mitochondrial ribosomal proteins that were observed in multiple bands were compared. To increase the data quality, proteins with a Mascot score lower than 45 were excluded from the list.

Ribosomal proteins were assigned to subunits according to their abundance in 28S and 39S fractions. The Exponentially Modified Protein Abundance Index (emPAI) score was calculated to compare the protein abundance in each sample (Ishihama et al., 2005). In brief, the ratio of the unique parent ion number observed in the analysis to the observable peptide number from *in silico* digestion is used as PAI in the formula: emPAI = 10^(PAI)−1^. The emPAI values were used to determine subunit distribution of each protein identified in 28S, 39S, and 55S samples.

### Preparation of crude ribosomes from human cell lines and isolated mitochondria

HeLa cells were grown in Dulbecco's Modified Eagle's Medium (DMEM) media (Cellgro, Mediatech Inc.) supplemented with 10% (v/v) bovine calf serum (Hyclone Laboratories) and 100 IU/ml penicillin and 100 μg/ml streptomycin at 37°C and 5% CO_2_ in a humidified atmosphere. For the whole cell lysate preparation, approximately 4 × 10^7^ HeLa cells were combined and lysed in 2 mL of buffer containing 50 mM Tris-HCl, pH 7.6, 0.26 M sucrose, 60 mM KCl, 20 mM MgCl_2_, 0.8 mM EDTA, 2 mM DTT, 0.05 mM spermine, 0.05 mM spermidine, 1.6% Triton X-100, and protease inhibitor cocktail from Sigma-Aldrich using a Dounce homogenizer (Wheaton). In order to isolate mitochondria, approximately 2 × 10^7^ HeLa cells were resuspended in 1 mL of an isotonic mitochondrial buffer (MB) (210 mM mannitol, 70 mM sucrose, 1 mM EDTA, 10 mM HEPES-KOH pH 7.5), supplemented with protease inhibitors (1 mM PMSF and the protease cocktail from Sigma-Aldrich described above), and then homogenized in a Dounce homogenizer on ice. The suspension was centrifuged at 400 × g in a microcentrifuge (ThermoForma) at 4°C. The pellet was resuspended in another 1 mL of MB and the 400 × g centrifugation was repeated. Supernatants were combined and centrifuged at 10,000 × g at 4°C for 10 min to pellet mitochondria. The mitochondrial pellets were lysed in a buffer containing 0.26 M sucrose, 20 mM Tris-HCl, pH 7.6, 40 mM KCl, 20 mM MgCl_2_, 0.8 mM EDTA, 0.05 mM spermine, 0.05 mM spermidine, 6 mM β-mercaptoethanol, and 1.6% Triton X-100 using a Dounce homogenizer.

To collect the crude ribosomes, whole cell and mitochondrial lysates (2 mL) were layered onto a 34% sucrose cushion (4 mL) in buffer (50 mM Tris-HCl, pH 7.6, 60 mM KCl, 20 mM MgCl_2_, and 6 mM β-mercaptoethanol) and centrifuged in a Type 40 rotor (Beckman Coulter) at 40,000 rpm for 16 h. The post-ribosomal supernatant was fractionated into six separate layers (designated L1–L6) for analysis, and the pellet was collected as a crude ribosomal fraction. The crude ribosome preparations, which included mitochondrial and cytoplasmic ribosomes for whole cell lysates and only mitochondrial ribosomes for the mitochondrial lysates, were resuspended in 50 μL of Base Buffer III (50 mM Tris-HCl, pH 7.6, 60 mM KCl, 20 mM MgCl_2_, 1 mM DTT) and protease inhibitor cocktail (Sigma-Aldrich). Ribosome suspensions were stored at −80°C for further analyses.

### RNase a treatment of mitochondrial ribosomes

In order to confirm the direct or indirect interaction of new MRPs with the rRNA of the mitochondrial ribosome, approximately ~5 A_260_ units of a crude preparation of ribosomes obtained from bovine liver were incubated in the absence or presence of 20 μ g RNase A and loaded onto separate 10–30% linear sucrose gradients in buffer containing 40 mM KCl, 20 mM MgCl_2_, 50 mM Tris-HCl, pH 7.6, and 1 mM DTT. After centrifugation, the proteins in equal volumes (25 μ L) of gradient fractions were separated on 12% SDS-PAGE. The proteins were transferred to PVDF membranes and probed with corresponding antibodies as described below.

### Immunoblotting

Ribosome samples collected from HeLa cell and bovine mitochondria (including sucrose gradient fractions, purified 55S ribosomes, 28S subunits, and 39S subunits) were separated by 12% SDS-PAGE. Proteins were transferred to PVDF membranes, which were probed with rabbit polyclonal anti-CHCHD1 antibody at a 1:1000 dilution (Abcam), rabbit anti-AURKAIP1 antibody at a 1:1000 dilution (Sigma-Aldrich), goat anti-CRIF1 antibody at a 1:500 dilution (Santa Cruz Biotechnology), mouse monoclonal anti-MRPS29 (DAP3) and anti-HSP60 antibodies at 1:5000 dilutions (BD Transduction Laboratories), mouse anti-OXPHOS (MITOPROFILE®) at a 1:5000 dilution (Abcam Inc.), or rabbit polyclonal human anti-MRPL47 and mouse polyclonal human anti-MRPS18-2 antibodies at 1:5000 dilutions (produced in-house) for 16 h at 4°C. All of the secondary antibodies were used at 1:5000 dilutions, including donkey anti-goat IgG (Santa Cruz Biotechnology) for CRIF1; goat anti-mouse IgG (Pierce Biochemicals Inc.) for MRPS29, HSP60 and OXPHOS, and goat anti-rabbit IgG for CHCHD1, AURKAIP1, and MRPL47. The membranes were developed using SuperSignal West Pico Chemiluminescent substrate (Pierce Biochemicals Inc.) according to the protocol provided by the manufacturer.

### [^35^S]-methionine pulse labeling of mitochondrial translation products *in vivo*

Human embryonic kidney 293T (HEK293T) cell lines were cultured in DMEM (Cellgro, Mediatech Inc.) supplemented with 10% bovine calf serum (Hyclone), 100 IU/ml penicillin, and 100 μg/ml streptomycin at 37°C and 5% CO_2_ in a humidified atmosphere. Cells were transfected with control siRNA (sc-44235) and mixtures of two to five target-specific siRNAs against CHCHD1 (sc-90488), AURKAIP1 (sc-72472), and CRIF1 (sc-97804) from Santa Cruz Biotechnology using Lipofectamine™ 2000 (Invitrogen) according to the protocol provided by the manufacturer. The transfected cells were grown in transfection medium for 2 days prior to labeling with [^35^S]-methionine. It should be noted that the siRNA treated cells grew very poorly reflecting the importance of CHCHD1, AURKAIP1, and CRIF1 for cell growth and viability and suggesting that they play an essential role in the cell. Labeling experiments were performed in the presence of dialyzed serum (25 mM Tris-HCl, pH 7.4, 137 mM NaCl, and 10 mM KCl) and minimum essential DMEM medium, which does not contain methionine, glutamine, or cysteine, as indicated in our previous reports following the protocol by Chomyn et al., with several modifications described by Leary et al. (Chomyn, 1996; Leary and Sasarman, 2009; Yang et al., 2010). Cells were incubated with emetine-containing medium for 5 min to arrest cytosolic protein synthesis, and 0.2 mCi/mL of [^35^S]-methionine (Perkin Elmer) containing medium was then added to label the mitochondrially-encoded proteins. After a 2 h incubation, cells were lysed in buffer containing 50 mM Tris-HCl, pH 7.6, 150 mM NaCl, 1 mM EDTA, 1 mM EGTA, 0.1% SDS and 0.5% NP-40 supplemented with 1 mM PMSF and protease inhibitor cocktail (Sigma-Aldrich). Whole cell lysates (40 μ g) were electrophoresed through 12% SDS-PAGE. The gels were dried on 3MM chromatography paper (Whatman), and the total intensities of the signals were quantified by phosphorimaging analyses (Jeffreys and Craig, 1976). The siRNA mediated knock-down efficiency of the corresponding mitochondrial ribosomal protein was confirmed with immunoblotting analysis of whole cell lysates or crude ribosomal fractions, prepared as stated above.

### Reverse transcription polymerase chain reaction (RT-PCR)

Total RNA was isolated from siRNA transfected HEK293T cells by using RNeasy Mini Kit from Qiagen. These RNA preparations were tested for mtDNA contamination using a minus reverse transcriptase control and shown to be free of mtDNA (data not shown). The cDNA was synthesized using the ThermoScript™ RT-PCR system (Invitrogen). The primers used were: CHCHD1 forward 5′- ACCTCTCATTCTAGCTAACCGCGT -3′, reverse 5′- AGACTCTCCCAGGGTTTCCTGTAT -3′; AURKAIP1 forward 5′- TCCACCGCAATCCTACCAGTGT -3′, reverse 5′- CGAACTTGATCTGCTTGCGTCTCA -3′; CRIF1 forward 5′- GATGATTGTGAACTGGCAGCAGCA -3′, reverse 5′- CGCCTCCTTCTTCCGTTTCTGTTT -3′; ND6 forward 5′- GAGTGTGGGTTTAGTAATGGGGTTTGTGGGG -3′, reverse 5′- CCTATTCCCCCGAGCAATCTC -3′; COI forward 5′- ATTTAGCTGACTCGCCACACTCCA -3′, reverse 5′- TAGGCCGAGAAAGTGTTGTGGGAA -3′; COII forward 5′- ATGGCACATGCAGCGCAAGTA -3′, reverse 5′- CTATAGGGTAAATACGGGCCC-3′; ATP6 forward 5′- TAATACGACTCACTATAGATGAACGAAAATATGT -3′, reverse 5′- TTTTTTTTTTTTTTTTTTTTTTCATTGTTGGGTGGTGATTAG -3′; 12s rRNA forward 5′- AATAGGTTTGGTCCTAGCCTAGCC -3′, reverse 5′- GTTCGTCCAAGTGCACTTTCCAG -3′; MRPS29 forward 5′- ATGGACCGACACGGGTATTGTACC -3′, reverse 5′- AAGGCCATGGGGAAATACAGTC -3′; MRPL47 forward 5′- AAACGGGGTACCGAGATGGCTGCGGCCGGTTTGGCCC -3′, reverse 5′- CCGCTCGAGTTAATGGTGATGGTGATGATGGACAAGACTTGACTTTTGGGC -3′; glyceraldehyde 3-phosphate dehydrogenase (GAPDH) forward 5′- GTCTTCACCACCATGGAGAAGG -3′, reverse 5′- ATGAGGTCCACCACCCTGTTGC -3′. Reactions were performed according to the instructions provided by the manufacturer. Samples were visualized using the ChemiDoc XRS system, employing Quantity One® 1D analysis software (Bio-Rad). The signal intensities obtained for control and siRNA knock-down samples were normalized to the GAPDH signals for the relative quantitation of RNA expression.

## Results and discussion

### Identification of 55S ribosomal proteins by tandem mass spectrometry

**Figure 1:**
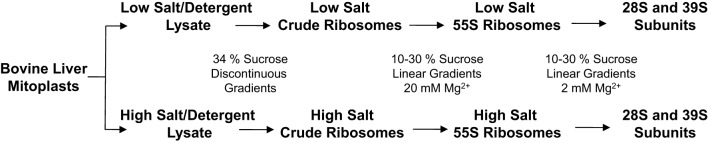
**Experimental scheme for the purification of mitochondrial ribosomes and 28S and 39S subunits**. To obtain purified 55S ribosomes and 28S and 39S subunits using sequential linear sucrose gradients, bovine mitochondrial lysates were prepared at different ionic and detergent conditions to remove mitochondrial metabolic enzymes co-sedimenting with the mitochondrial ribosome. Experimental details are included in Materials and Methods.

The majority of the protein components of mammalian mitochondrial ribosomes were identified by our group, as well as several other groups, a decade ago using proteomics strategies (Goldschmidt-Reisin et al., 1998; Graack et al., 1999; Koc et al., 1999, 2000, 2001a,b; O'Brien et al., 1999). However, due to the limited availability of bovine or rat protein and DNA sequence information, some of the ribosomal and ribosome-associated proteins were not detected or identified by matching the tandem MS data to the publicly available ESTs or protein databases available at the time. An analysis of the protein composition of a large macromolecular complex requires the preparation of that complex under conditions that are strong enough to remove contaminants, but that are gentle enough to prevent the loss of more loosely bound protein components. In order to re-evaluate their protein composition and to eliminate transiently associated proteins, mammalian mitochondrial ribosomes were purified under two different salt and detergent conditions from bovine liver (Figure 1) (Spremulli, 2007). The 10–30% linear sucrose gradient separation of mitochondrial ribosomes was carried out at 20 mM Mg^2+^ to collect 55S particles (Figure 1).

The protein compositions of the 55S samples prepared under the two different salt and detergent conditions were compared by SDS-PAGE (Figure S1). In general, the gel pattern was similar in both ribosomal preparations; however, the sample prepared at high salt and detergent concentrations contained relatively lower amounts of high molecular weight proteins. The proteins in these bands may be subunits of the large enzyme complexes that co-sediment with the mitochondrial ribosome fractions (Figure S1). The gels were sliced into thirty fractions, and the in-gel tryptic digestion of each gel piece was analyzed by capLC-MS/MS. The peptide and protein contents of the gels were determined by matching MS/MS spectra to an in-house protein sequence database, which was generated by the combination of ~30,000 bovine proteins found in the Swiss-Prot and UniProtKB databases.

A comprehensive list of the MRPs, the subunits of metabolic enzyme complexes, and the other proteins identified in our analyses is given in Tables S1 and S2. The list of the proteins that had not previously been identified as *bona fide* ribosomal proteins was created by excluding the known MRPs, mitochondrial metabolic pathway proteins, and oxidative phosphorylation proteins. In these analyses, CHCHD1, AURKAIP1, PTCD3, and CRIF1 were repeatedly found to be present in the mitoribosome with high confidence in both low and high salt and detergent preparations (Tables 1, S2, S3). It should be noted that CRIF1 and PTCD3 were known from previous studies to be associated with the ribosome (Davies et al., 2009; Kim et al., 2012). ICT1 (MRPL58) was also observed in these studies and was reported previously as a mitochondrial ribosomal protein (Richter et al., 2010). With the exception of large complexes of metabolic enzymes (specifically 2-oxoglutarate and pyruvate dehydrogenases and ATP synthase F1 subunits sedimenting with the ribosome), proteins consistently found in both low and high salt and detergent preparations of 55S ribosomes were considered as possible components of the mitochondrial translational machinery and ribosomal proteins (Tables 1, S1).

**Table 1:** **Peptide sequences of new mitochondrial ribosomal proteins identified from LC-MS/MS analyses of in-gel tryptic digestions of 28S, 39S, and 55S samples prepared at high salt conditions**.

**Name**	**Sequence**	**Score**	**m/z**	**Mr (expt)**
CHCHD1	KPILKPNKPLILANHVGER	97	713.2	2136.7
	SIQEDLGELGSLPPR	112	805.7	1609.4
	SIQEDLGELGSLPPRK	85	870.4	1738.7
AURKAIP1	AGLKEAPPGWQTPK	74	740.7	14.79.4
	EAPPGWQTPK	83	556.7	1109.6
PTCD3	DEGADIAGTEEVVIPK	113	821.7	1641.5
	TWDKVAVLQALASTVHR	145	948.4	1894.9
	VAVLQALASTVHR	88	683	1364.1
	AGHQLGVTWR	63	563.4	1124.7
	AHTQALSMYTELLNNR	143	931.9	1861.7
	ADVHTFNSLIEATALVVNAK	150	1057.8	2113.7
	WNNILDLLK	54	564.9	1127.9
	QMVAQNVKPNLQTFNTILK	110	1094.5	2186.9
	GSSLIIYDIMDEITGK	118	878.3	1754.6
	FSPKDPDDDMFFQSAMR	72	723.6	2167.9
	DPDDDMFFQSAMR	102	788.2	1574.4
	DLELAYQVHGLLNTGDNR	124	1014.9	2027.9
	LEMIPQIWK	55	579.4	1156.8
	SDLKEEILMLMAR	91	775.4	1548.9
	EEILMLMAR	51	553.6	1105.2
	NELLNEFMDSAK	80	706.3	1410.5
	ASSSPAQAVEVVK	79	636.7	1271.4
	LTADFTLSQEQK	95	691	1379.9
	EALGDLTALTSDSESDSDSDTSKDK	90	863.8	2588.4
ICT1	QGNDDIPVDR	90	565.3	1128.5
	AGELILTSEYSR	79	670.4	1338.8
	GADTAWRVPGDAK	60	672.6	1343.2
	SAYSLDKLYPESR	95	766.3	1530.7
	FHLASADWIAEPVR	119	806.8	1611.5
	VPGDAKQGNDDIPVDR	69	565.6	1693.9
	DMIAEASQPATEPSKEDAALQK	124	1165.9	2329.9
CRIF1	HGAASGVDPGSLWPSR	115	797.5	1593.1
	AAAMAAAAAQDPADSETPDS	116	930.9	1859.8
	EQLLELEAEER	77	680.3	1358.6
	MPQMIENWR	54	602.9	1203.8
	FQELLQDLEK	62	632.3	1262.7

### Subunit assignments of ribosome-associated proteins by LC-MS/MS

**Figure 2:**
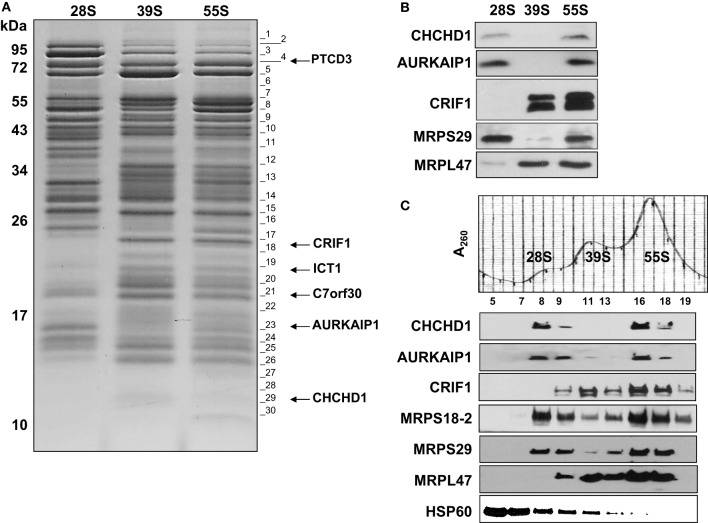
**Identification and detection of mitochondrial ribosomal proteins and their subunit association by capLC-MS/MS and immunoblotting analyses. (A)** Purified mitochondrial (55S) ribosomes (~10 A_260_) prepared in high salt conditions were dissociated into subunits by sedimentation through a second 10–30% linear sucrose gradient in the presence of 2 mM Mg^2+^ as described in Figure 1. After the sedimentation of the purified subunits, the same A_260_ units of 28S and 39S subunits and 55S ribosomes (~0.5) were separated on SDS-PAGE. The gel was cut into 30 pieces for in-gel digestion of protein bands with trypsin and the peptides were analyzed by capLC-MS/MS analysis to identify the proteins present. Gel fractions containing the peptides detected from newly identified MRPs are marked on the image. Proteins identified in these gel pieces are given in Tables S1, S2. **(B)** Purified 28S and 39S subunits (~0.2 A_260_) were obtained from the dissociation of purified 55S ribosomes as described in Materials and Methods at 2 mM Mg^2+^. Proteins present in these preparations were separated on 12% SDS-PAGE and analyzed by immunoblotting using CHCHD1, AURKAIP1, CRIF1, MRPS29, and MRPL47 antibodies to confirm subunit distribution of new MRPs. **(C)** To confirm the ribosome association of newly identified proteins, crude mitochondrial ribosomes prepared at high salt and detergent conditions were separated on a 10–30% linear sucrose gradient in the presence of 20 mM Mg^2+^ as described in Figure 1. Distribution of new proteins, CHCHD1, AURKAIP1, and CRIF1, were detected by immunoblotting analyses of sucrose gradient fractions containing dissociated ribosomal subunits (28S and 39S) and 55S ribosomes. Antibodies against two 28S subunit proteins, MRPS18B and MRPS29, and a 39S protein, MRPL47, were used to indicate the locations of the subunits and the 55S ribosome.

In order to categorize the newly observed proteins as either MRPs or proteins involved in translation, determining the subunit assignments of these proteins was essential. Sucrose gradient purified bovine 55S ribosomes prepared at high salt and detergent conditions (Figure S1) were sedimented on a second 10–30% linear sucrose gradient containing 2 mM Mg^2+^ to promote dissociation of the small (28S) and large (39S) subunits (Figure 2A). Proteins contained in the purified 28S, 39S, and 55S samples were separated by SDS-PAGE and stained with Coomassie Blue (Figure 2A). Although the amount of high molecular weight proteins found in the 55S ribosome preparation decreased, several remained associated with the 28S and 39S subunits in the second sucrose gradient (Figure 2A). The Coomassie Blue stained gel clearly showed the differential protein and MRP distribution in the 28S and 39S fractions, and the 55S fraction contains the protein bands observed in both 28S and 39S fractions (Figure 2A). Each lane was cut into thirty equal pieces. In-gel tryptic digestions were carried out, and the resulting peptides were analyzed by capLC-MS/MS. Database searches were performed as described for the analysis of 55S ribosomal proteins.

In general, the same set of proteins found in the 55S ribosomes was also identified in 28S and 39S subunits (Table S1). This table provides a complete list of the MRPs and the newly identified proteins, as well as their distributions in the 28S and 39S subunits. The proteins transiently associated with the 55S ribosome (including mitochondrial elongation factor EF-Tu, ribosome recycling factor (RRF), and a bacterial yBEB homolog, C7orf30) were released during either the 55S ribosome preparation or the dissociation of the ribosome into 28S and 39S subunits in the second sucrose gradient at 2 mM Mg^2+^ (Table S1). Four additional proteins not previously assigned as mitochondrial ribosomal proteins (CHCHD1, AURKAIP1, PTCD3, and CRIF1) were consistently observed in 28S or 39S subunits and in 55S ribosomes, making the determination of whether these proteins are *bona fide* ribosomal proteins or ribosome-associated proteins critical. The relative molecular masses of these proteins were in good agreement with their expected molecular weights after the removal of mitochondrial import signals (Figure 2A) (Claros and Vincens, 1996). The Mascot scores obtained from database analyses of these peptides revealed that these proteins have clear subunit distributions in either the 28S or 39S subunit, except for ICT1 (Table S1). In addition to the Mascot scores obtained for each protein, the experimental emPAI scores determined as described by Ishihama et al. (Ishihama et al., 2005) were calculated to demonstrate the relative protein abundance in each subunit, using the ratio of peptides detected by capLC-MS/MS analyses to the number of observable peptides obtained from *in silico* digestion of a protein (Tables 2, S1, S3). The GenBank™ and Swiss-Prot access numbers of the newly identified MRPs used in the emPAI determination are listed in Table 3. The agreement between the emPAI scores for the 55S ribosome and either the 28S or 39S values for each protein clearly shows that these proteins were associated with the ribosome and its subunits under the conditions used in our experiments (Table 2). Variations in emPAI scores, specifically for PTCD3 and ICT1, could be due to discrepancies in the excision of gel slices, and the extraction of peptides in different samples, or the data dependent acquisition of peptides by the capLC-MS/MS system.

**Table 2:** **Relative distribution of new mitochondrial ribosomal proteins using emPAI (Exponentially Modified Protein Abundance Index) values calculated from peptides detected by LC-MS/MS analyses of proteins in 28S and 39S subunits and 55S ribosomes**.

**Subunit**	**CHCHD1**	**AURKAIP1**	**PTCD3**	**ICT1**	**CRIF1**
28S	3.30	0.27	169.61	0.97	0.49
39S	ND	0.19	0.64	1.09	2.02
55S	3.14	0.27	22.77	0.86	2.11

Peptides (ion score cut off of ≥45) listed in Table S3 were used in emPAI calculations. ND; not detected.

**Table 3:** **GenBank™ and Swiss-Prot accession numbers of new mitochondrial ribosomal proteins found in various species**.

**MRP**	**Protein**	**Human**	**Bovine**	**Mouse**	**Fly**	**Worm**
MRPS37	CHCHD1	Q96BP2	Q2HJE8	XP_852408	ABJ16982	AAB88317
MRPS38	AURKAIP1	Q9NWT8	Q0VCJ1	XM_843641	Q8IML6	Y54G9A
MRPS39	PTCD3	Q96EY7	Q2KI62	XP_532975	A1Z9A8	AAF60413
MRPL58	ICT1	Q14197	Q3T116	XP_533118	CAL26738	AAL06045
MRPL59	CRIF1	Q8TAE8	A1A4P4	XP_533898	AAM29650	CAB03171

To confirm the subunit distribution determined by the emPAI scores, immunoblotting analyses of purified subunits and 55S ribosomes were performed using CHCHD1, AURKAIP1, and CRIF1 antibodies. Previous work had indicated that PTCD3 was associated with the small subunit, while CRIF1 and ICT1 interacted with the large subunit (Koc and Spremulli, 2003; Richter et al., 2010; Haque et al., 2011; Kim et al., 2012). In the present analyses, CHCHD1 and AURKAIP1 signals overlapped with the MRPS29 signals, which were clearly detected in 28S and 55S fractions, indicating their association with the small subunit. CRIF1 was detected in 39S and 55S preparations, along with the large subunit protein MRPL47 (Figures 2B,C). The data obtained by capLC-MS/MS and immunoblotting analyses cooperatively suggest that CHCHD1 and AURKAIP1 are newly described components of the small subunit. Further, the data indicate that CRIF1 is not only associated with the large subunit, but also a *bona fide* component of the large subunit of the mitochondrial ribosome. Alignments of new MRPs across worm, fly, mouse, bovine, and human homologs (as well as yeast homologs of CHCHD1 and AURKAIP1) indicate the presence of evolutionarily conserved regions in these new proteins (Figure S2). Some of the characteristics of these new ribosomal proteins are described below.

#### CHCHD1

Although a signal peptide cleavage site was not predicted by MitoProt II, the probability of a mitochondrial localization of bovine CHCHD1 is 81% (Claros and Vincens, 1996). The accession numbers for CHCHD1 from various organisms, used in the alignment of the full length sequences, are given in Table 3 (Figure S2). The full length bovine CHCHD1 is about 13.6 kDa. Mass spectrometry analysis of tryptic peptides extracted from the protein bands excised from 28S, 39S, and 55S lanes resulted in identification of CHCHD1 peptides in 28S and 55S samples, but not in 39S subunits (Tables 1, S1). Mammalian mitochondrial CHCHD1 has a homolog in the yeast mitochondrial ribosome, MRP10, as reported by Smits et al., which has about 20% sequence identity to the human protein (Graack et al., 1992; Jin et al., 1997). Similarly, the fly and worm homologs of CHCHD1 are also 20–25% identical to mammalian homologs, although their sequence identity to the yeast homolog is below 14% (Figure S2). The emPAI values calculated using CHCHD1 peptides clearly suggest that this protein is mainly associated with the small subunit of mitochondrial ribosomes (Tables 2, S3). The data presented here provide the first experimental evidence indicating that CHCHD1 is a component of the small subunit in mammalian mitochondria.

#### AURKAIP1

**Figure 3:**
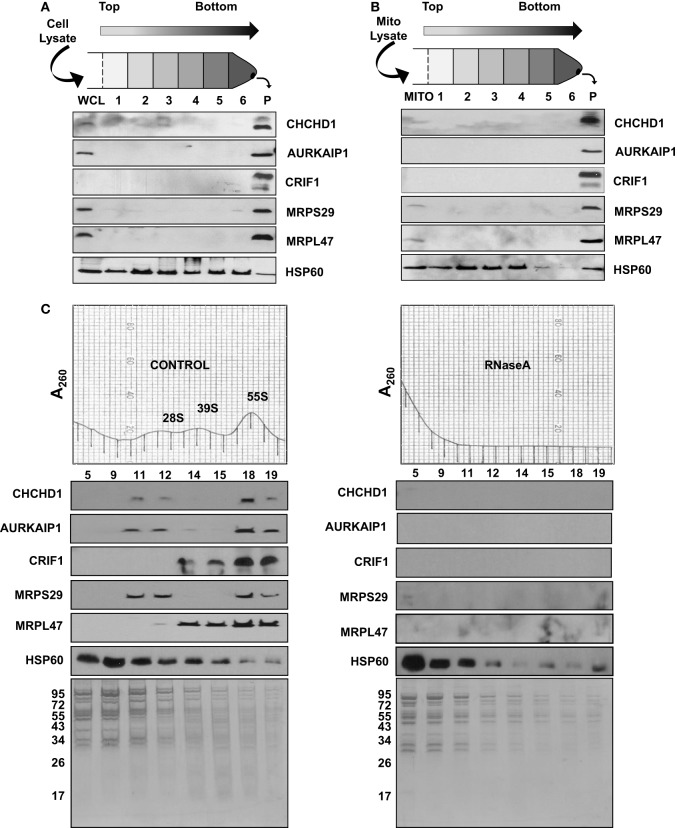
**Sedimentation of new MRPs with large complexes in human cell lines and mitochondria. (A)** Whole cell lysate (WCL) and **(B)** mitochondrial lysate (MITO) obtained from human cell lines were layered on sucrose cushion preparations. After 16 h centrifugation, post-ribosomal supernatant layers (Layers 1 to 6) and the crude ribosome pellet (P) were collected and analyzed by immunoblotting probed with CHCHD1, AURKAIP1, CRIF1, MRPS29, MRPL47, and HSP60 antibodies. **(C)** To confirm the RNA-dependent association of new MRPs with mitochondrial ribosomes, the same amount of crude ribosomes, both Control and RNase A-treated, were sedimented on 10–30% sucrose gradients. Equal volumes of sucrose gradient fractions were separated on 12% SDS-PAGE. Sedimentation of new MRPs with ribosomal subunits and 55S ribosomes in the absence and presence of RNase A was analyzed by immunoblotting using CHCHD1, AURKAIP1, CRIF1, MRPS29, and MRPL47 antibodies. HSP60 immunoblotting and the Coomassie Blue stained gel are shown as controls for multimeric mitochondrial complexes sedimenting with the 55S ribosome in control and RNase A-treated gradients.

AURKAIP1, also known as Aurora-A-interacting protein (AIP), was first described as a regulator of Aurora-A kinase, which is a Ser/Thr kinase involved in cell cycle progression and tumorigenesis (Kiat et al., 2002). The homology region found in AURKAIP1 homologs is termed DUF1713, and this region is found in the C-terminal domain of yeast COX24 (Figure S2). Yeast COX24 was described as one of the factors responsible for COI mRNA processing and translation (Barros et al., 2006). The calculated molecular mass of the full-length AURKAIP1 is 22.4 kDa; however, the mature protein migrates at about 16 kDa (Figures 2B, 3). It is possible that AURKAIP1 has a longer signal peptide than the predicted signal peptide, which provides a 99% likelihood for the translocation of this protein into mitochondria. Translocation of AURKAIP1 into the mitochondria has also been experimentally validated by the Human Protein Atlas (HPA) project (Uhlen et al., 2010). AURKAIP1 was repeatedly detected in the capLC-MS/MS analyses of low and high salt preparations of bovine mitochondrial ribosomes and their subunits. The calculated emPAI values for peptides obtained from purified 28S and 39S preparations suggest the association of AURKAIP1 with the small subunit, considering that the emPAI value for the 28S subunit is 30% higher than that of the 39S subunit (Tables 1, 2, S1, S3).

#### PTCD3

We first reported PTCD3 as an mRNA-binding protein associated with the 28S subunit and as a possible PET309 homolog, due to the presence of pentatricopeptide repeats (Koc and Spremulli, 2003). The association of PTCD3 with the small subunit and its essential role in mitochondrial translation has also been reported (Davies et al., 2009). Our data not only confirms the association of PTCD3 with the small subunit, but also provides evidence that it is a *bona fide* ribosomal protein. This idea was recently reinforced by the observation that PTCD3 is one of the 28S subunit proteins that interacts with mitochondrial initiation factor 3 (Haque et al., 2011). The full-length bovine PTCD3 is 77.8 kDa with a pI of 6.0; it is possibly the largest protein, and one of the most acidic components of the 55S ribosome. It is highly conserved among its animal mitochondrial homologs (Figure S2). PTCD3 is a larger protein and has a lower pI compared to the basic proteins of the mitochondrial ribosome, and nineteen unique peptides were detected in its capLC-MS/MS analyses (Tables 1, S3). In agreement with earlier observations, PTCD3 peptides were mainly detected in protein bands excised from 28S and 55S lanes (Figure 2A and Table S3). Its subunit localization is also supported by the emPAI values shown in Table 2; specifically, the high experimental emPAI value for peptides identified in the 28S subunit sample, as well as in the 55S sample, indicates that PTCD3 is a ribosomal component associated with both the 28S subunit and 55S ribosomes (Tables 2 and S3). The presence of a trace amount of PTCD3 in 39S preparations could be due to the presence of a small amount of 28S subunits in the large subunit fractions analyzed. This cross-contamination is common because there is a tendency for the 55S ribosome to dissociate somewhat during sucrose gradient centrifugation (Koc et al., 2001b). The high emPAI scores of the 28S subunit and 55S ribosomes enabled us to conclude that PTCD3 is a mitochondrial-specific MRP that is mainly associated with the 28S subunit.

#### ICT1

Our data confirm previous reports that ICT1 is a mitochondrial ribosomal protein. This protein belongs to the polypeptide release family of proteins, and it is the first example of an integral component of the large subunit providing peptidyl-tRNA hydrolase activity (Richter et al., 2010). ICT1 is essential for the hydrolysis of peptidyl-tRNAs on prematurely terminated mRNAs that lack a stop codon (Handa et al., 2010; Richter et al., 2010). It is a highly conserved protein among animals (Figure S2). Tryptic peptides detected by capLC-MS/MS analyses of protein bands are listed in Tables 1 and S3. ICT1 peptides were detected in 28S, 39S, and 55S samples, which made the calculated emPAI values of the ICT1 in 28S and 39S subunits very close to each other (Table S3). The experimental emPAI scores were not sufficiently different to assign ICT1 to a particular subunit (Table 2). The human ICT1 was previously shown to be associated with the large subunit (Richter et al., 2010); however, our MS data do not clearly support association of ICT1 with the large subunit (Table 2). It is reasonable to suggest that ICT1 is located near the interface region of the large subunit, and that a fraction of ICT1 also sediments with the small subunit when the subunits are dissociated.

#### CRIF1

CR-6 interacting factor 1 (CRIF1), which is also known as growth arrest and DNA-damage-inducible proteins-interacting protein 1 (Gadd45GIP1), was identified as a transcriptional co-activator. The calculated molecular mass of the full-length bovine CRIF1 is 25.7 kDa. Its mitochondrial localization signal peptide is predicted to be in the first 28 amino acid residues, and it has a 90% possibility of mitochondrial localization (Figure S2). The calculated molecular mass for the mature CRIF1 is 22.9 kDa, and this value is in agreement with the migration of the protein in SDS-PAGE detected by capLC-MS/MS (Figure 2A). The emPAI values, calculated from five unique CRIF1 peptides detected in 39S and 55S samples, strongly suggest that the mitochondrial CRIF1 is a large subunit protein (Tables 2 and S3).

### Subfractionation of CHCHD1, AURKAIP1, and CRIF1 with mitochondrial ribosomes

In the capLC-MS/MS analyses of highly purified bovine mitochondrial ribosomes and subunits presented above, we were able to assign the subunit in which the newly identified ribosomal proteins were present. Association of PTCD3 and ICT1 with the mitochondrial ribosome was reported in our earlier studies and by other groups (Koc and Spremulli, 2003; Richter et al., 2010). However, we are describing and characterizing CHCHD1, AURKAIP1, and CRIF1 as integral components of the mitochondrial ribosome for the first time. Ectopically expressed forms of CRIF1 and AURKAIP1 have been localized to the nucleus and cytosol and reported to be involved in the transcriptional activation and regulation of Aurora-A kinase in human cell lines, respectively (Kang et al., 2010). In order to evaluate their association with the mitochondrial ribosome, human whole cell and mitochondrial lysates were prepared under non-denaturing conditions and centrifuged through a 34% sucrose cushion, a step analogous to that used in the preparation of bovine liver mitochondrial ribosomes (Figures 3A,B). This process enriched the multimeric complexes resistant to high salt and non-ionic detergent treatments in the bottom layer and pellet, mainly as cytoplasmic 80S and mitochondrial 55S ribosomes. In immunoblotting analyses of post-ribosomal supernatant layers and crude ribosomal pellets obtained from the whole cell (Figure 3A) and mitochondrial lysates (Figure 3B), these MRPs were found mainly in the pellet along with two mitochondrial ribosomal proteins (MRPS29 and MRPL47), thus, confirming their co-localization with the mitochondrial ribosome or other large complexes in mitochondria.

Immunoblotting analyses and identification of these proteins in highly purified ribosomal subunits, 55S ribosomes (Figure 2), and crude ribosomes (Figure 3B) support their ribosome association; however, it is possible that these proteins could be associated with the large mitochondrial complexes that co-sediment with the ribosome but have nothing to do with mitochondrial translaiton. For example, we have observed that polypeptides of the oxoglutarate dehydrogenase complex can be detected in the 28S region of the gradient. To demonstrate that the proteins under study here are not simply co-sedimenting with the ribosome but are actually associated with the particle, RNase A-treatment of the 55S ribosome was carried out prior to loading the samples onto the sucrose gradients and immunoblotting analyses. RNase A-treatment destroys the integrity of the mitochondrial ribosome (Figure 3C), as it does other ribosomes, as indicated by the loss of the A_260_ from the 55S region of the sucrose gradient following nuclease treatment and the increase in the A_260_ near the top of the gradient. As shown in Figure 3C, the signals from the new MRPs overlapped with those from MRPS29 and MRPL47 in the control sucrose gradient fractions. When the ribosome sample was treated with RNase, signals from CHCHD1, AURKAIP1, and CRIF1 disappeared from the 55S region of the gradient as did the signals from the two control proteins, MRPS29 and MRPL47. This data indicates that these proteins are either assembled into the ribosome or strongly associated with the mitoribosome and are not simply co-fractioning with the 55S particles on the sucrose gradient. On the other hand, the HSP60 signal remained unchanged in these fractions, along with high molecular weight proteins which were subunits of the other multimeric complexes of mitochondria detected by Coomassie Blue stained SDS-PAGE (Figure 3C).

### Role of CHCHD1, AURKAIP1, and CRIF1 in mitochondrial translation

**Figure 4:**
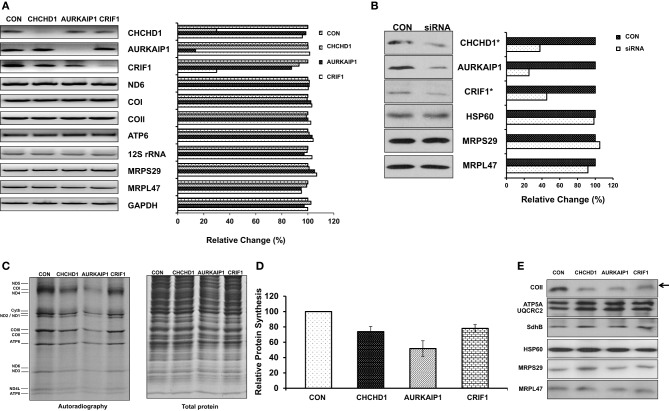
**Role of CHCHD1, AURKAIP1, and CRIF1 knock-down on mitochondrial protein synthesis. (A)** Transcript levels of CHCHD1, AURKAIP1, and CRIF1 were shown as an indication of knock-down efficiency in cell lines transfected with control siRNA and siRNAs for corresponding new MRPs. Mitochondrial encoded ND6, COI, COII, ATP6, and 12S rRNA, and nuclear encoded MRPS29 and MRPL47 were examined by RT-PCR reactions in the same samples. RT-PCR reactions with GAPDH were performed as a positive control and relative quantitation of signal intensities was calculated based on GAPDH mRNA levels (or siRNA control cell line) in each knock-down cell line. **(B)** Expression levels of new MRPs in control siRNA and MRP-specific siRNA transfected HEK293T cell lysates and total crude ribosome fractions (^*^) were analyzed by immunoblotting using antibodies against AURKAIP1, CHCHD1, CRIF1, MRPS29, MRPL47, and HSP60. Immunoblotting analyses with HSP60, MRPS29, and MRPL47 antibodies were used as loading controls for total protein and total ribosome content using whole cell lysates obtained from control and AURKAIP1 siRNA knock-down cells. Similar results were obtained with immunoblotting analyses of total crude ribosome fractions from CHCHD1 and CRIF1 siRNA knock-down cell lines using HSP60, MRPS29, and MRPL47 antibodies (data not shown). **(C)** *De novo* synthesis of mitochondrial proteins was evaluated in control siRNA and the siRNA of corresponding MRPs transfected HEK293T cells by pulse labeling of proteins in the presence of [^35^S]-methionine and a cytosolic translation inhibitor, emetine. A representative electrophoretic pattern of the *de novo* synthesized translational products is presented. ND1, −2, −3, −4, −4L, −5, and −6 are subunits of Complex I; Cyt*b* is a subunit of Complex III; COI, −II, and −III are subunits of Complex IV; ATP6 and ATP8 are subunits of Complex V. Coomassie blue staining of the same gel was performed to ensure equal protein loading in the gel. **(D)** The combined intensities of the 13 mitochondrially-encoded proteins from each lane were used as an overall quantitation of mitochondrial protein synthesis from three separate experiments. **(E)** Immunoblotting analysis of whole cell lysates prepared from control siRNA and MRP specific siRNA transfected HEK293T cells was performed to examine the steady state levels of the mitochondrial encoded subunit of Complex IV (COII) and nuclear encoded subunits of Complex V (ATP5A), III (UQCRC2), and II (SDHB). The arrow shows the reduced translation of mitochondrial encoded protein COII in cell lines transfected with CHCHD1, AURKAIP1, and CRIF1 specific siRNAs. The same PVDF was reprobed with MRPS29, MRPL47, and HSP60 antibodies to ensure equal loading.

To investigate the role of newly identified MRPs in mitochondrial translation, we first transfected the HEK293 cells using control and specific siRNAs corresponding to CHCHD1, AURKAIP1, and CRIF1 mRNAs. The siRNA knock-down efficiency was determined by RT-PCR analyses using total RNA isolated from each cell line. In addition to the effect of siRNA knock-down on CHCHD1, AURKAIP1, and CRIF1 mRNA expression, we also determined the changes in expression of several nuclear encoded MRPs and mitochondrially encoded genes (Figure 4A). In specific siRNA knock-down cells, CHCHD1 and CRIF1 mRNA levels were reduced by 70% of the control siRNA knock-down cells, whereas the reduction in AURKAIP1 mRNA was about 85% (Figure 4A). Clearly, none of the nuclear encoded MRP mRNAs or the representative mitochondrially encoded mRNAs (ND6, COI, COII, ATP6, and 12S rRNA) were affected by the reduction in CHCHD1, AURKAIP1, or CRIF1 mRNAs (Figure 4A). Treatment with the specific siRNAs lead to the reduced expression (from 55 to 75%) of the corresponding CHCHD1, AURKAIP1, and CRIF1 proteins, as confirmed by immunoblotting analyses of whole cell lysates and ribosomes enriched from knock-down cell lines using corresponding antibodies (Figure 4B). Since changes in the expression of the newly identified MRPs by siRNAs had no effect on the expression of mitochondrially encoded mRNAs and rRNAs, the newly identified proteins are not directly involved in transcription or RNA processing in mammalian mitochondria. Furthermore, the presence of normal amounts of the 12S rRNA indicates that there is no defect in the synthesis or assembly of the mitoribosome suggesting that CHCHD1, AURKAIP1, and CRIF1 are unlikely to be proteins involved in ribosome assembly.

Given the association of new MRPs with the mitochondrial ribosome, we next examined the role of CHCHD1, AURKAIP1, and CRIF1 knock-downs on the *de novo* synthesis of mitochondrially-encoded proteins by [^35^S]-methionine labeling in the presence of the cytosolic protein synthesis inhibitor emetine. In this analysis, only the thirteen essential subunits of the OXPHOS complexes synthesized by the mitochondrial translational machinery were labeled by [^35^S]-methionine (Figure 4C). In cells transfected with CHCHD1, AURKAIP1, and CRIF1 siRNAs, relative mitochondrial protein synthesis was decreased by 27, 48, and 22%, respectively, whereas the total protein content of the cell lysates was comparable (Figures 4C,D). Clearly, the inhibition of protein synthesis in knock-down cells was due to a reduction in expression of new MRPs, because reprobing the same membrane with MRPS29, MRPL47, and HSP60 antibodies displayed no significant difference in ribosomal or mitochondrial protein levels (Figure 4E). Although a considerable amount of new MRPs remained in siRNA knock-down cells (Figure 4B), the 22–48% reduction in overall mitochondrial translation is significant. In addition to the decrease in pulse labeling of mitochondrially encoded proteins, the steady state levels of the mitochondrially encoded subunit of Complex IV, COII, was also reduced in cells transfected with MRP-specific siRNAs, as detected by immunoblotting analysis (Figure 4E). On the other hand, the nuclear encoded subunits of oxidative phosphorylation complexes, such as Complex V subunit ATP5A, Complex III subunit Core II subunit (UQCRC2), and Complex II SDHB subunit, remained unchanged (Figure 4E). Altogether, these observations strongly suggest that CHCHD1, AURKAIP1, and CRIF1 all have essential roles in mitochondrial protein synthesis as components of the small and large subunits of the 55S ribosome.

### Nomenclature

The two-dimensional gel analysis of bovine mitochondrial ribosomes lead to the prediction of as many as 33 ribosomal proteins in the 28S subunit and 52 ribosomal proteins in the 39S subunit (Koc et al., 2001a,b). These proteins were designated S1 through S33 for the 28S subunit and L1 through L52 for the 39S subunit, in order of decreasing molecular weights (Pietromonaco et al., 1991). Later, we and several other groups identified 29 and 49 MRPs in the small and large subunits, respectively, using proteomic analyses (Goldschmidt-Reisin et al., 1998; Graack et al., 1999; Koc et al., 1999, 2001a,b; Suzuki et al., 2001; Yang et al., 2010). To provide consistency for designation of the same proteins in different organisms, we adopted a system of nomenclature in which proteins with prokaryotic homologues are given the same number as the corresponding ribosomal protein in *E. coli*. Proteins without bacterial homologs are given the next available number. Bacterial ribosomes have proteins designated S1 through S21 in the 28S subunit and L1 through L36 in the large subunit. Additionally, previously identified mammalian ribosomal proteins without bacterial homologs were designated S22 through S36 for the 28S subunit and L37 through L57 for the 39S subunit, including a new number for MRP63 (MRPL57), because its association with the 39S subunit was not previously known (Table 4) (Kenmochi et al., 2001). As a result, we proposed that the newly identified small subunit proteins (CHCHD1, AURKAIP1, and PTCD3) be named MRPS37, MRPS38, and MRPS39 and that ICT1 and CRIF1, as newly identified large subunit proteins, be designated as MRPL58 and MRPL59, respectively (Table 4). Here, the adaptation of the new MRP numbers is absolutely critical for their classification as mitochondrial ribosomal proteins and for further investigation of their roles in mitochondrial translation and biogenesis.

**Table 4:** **List of mammalian mitochondrial ribosomal proteins with their bacterial homologs**.

**28S proteins**	**30S proteins**	**New class**	**39S proteins**	**50S proteins**	**New class**	**50S proteins**
Missing	S1	MRPS22	MRPL1	L1	MRPL33	L33
MRPS2	S2	MRPS23	MRPL2	L2	MRPL34	L34
MRPS24	S3	MRPS24	MRP-L3	L3	MRPL35	L35
Missing	S4	MRPS25	MRPL4	L4	MRPL36	L36
MRPS5	S5	MRPS26	Missing	L5	MRPL37	
MRPS6	S6	MRPS27	Missing	L6	MRPL38	
MRPS7	S7	MRPS28	MRPL12	L7/L12	MRPL39	
Missing	S8	MRPS29	MRPL9	L9	MRPL40	
MRPS9	S9	MRPS30	MRPL10	L10	MRPL41	
MRPS10	S10	MRPS31	MRPL11	L11	MRPL42	
MRPS11	S11	MRPS32 (MRPL42)	MRPL13	L13	MRPL43	
MRP-S12	S12	MRPS33	MRPL14	L14	MRPL44	
Missing	S13	MRPS34	MRPL15	L15	MRPL45	
MRPS14	S14	MRPS35	MRPL16	L16	MRPL46	
MRPS15	S15	MRPS36	MRPL17	L17	MRPL48	
MRPS16	S16	MRPS37 (CHCHD1)	MRPL18	L18	MRPL49	
MRPS17	S17	MRPS38 (AURKAIP1)	MRPL19	L19	MRPL50	
MRPS18-1	S18	MRPS39 (PTCD3)	MRPL20	L20	MRPL51	
MRPS18-2	S18		MRPL21	L21	MRPL52	
MRPS18-3	S18		MRPL22	L22	MRPL53	
Missing	S19		MRPL23	L23	MRPL54	
Missing	S20		MRPL24	L24	MRPL55	
MRPS21	S21		Missing	L25	MRPL56	
			MRPL27	L27	MRPL57 (RP_63)	
			MRPL28	L28	MRPL58 (ICT1)	
			MRPL47	L29	MRPL59 (CRIF1)	
			MRPL30	L30		
			Missing	L31		
			MRPL32	L32		

### Potential roles of new mitochondrial ribosomal proteins in translation

Ribosomal proteins have roles in ribosome assembly, substrate binding, and different stages of translation, such as initiation, elongation, and termination. Furthermore, they are likely to interact with the nascent chain and facilitate their insertion into the respiratory chain complexes in the mitochondrial inner membrane. The MRPs with bacterial homologs are expected to have conserved functions in ribosome structure and translation. For the mammalian mitochondrial-specific proteins without known homologs in other ribosomes, it is highly challenging to identify and assign whether a ribosome-associated protein is a genuine ribosomal protein or a factor transiently associated with the ribosome. In fact, a recent bioinformatics survey of the evolutionarily conserved ribosomal proteins failed to identify several additional components of the mammalian mitochondrial ribosome (Smits et al., 2007). The criteria used in this study, however, allowed us to characterize the newly identified proteins as mitochondrial-specific ribosomal proteins. One issue that arises in these studies is to assess whether a protein observed in the ribosome preparations is a *bona fide* ribosomal protein or represents a protein transiently associated with the ribosome. Several criteria can be applied in making this determination. First, the newly assigned protein must have a distribution that parallels that of known ribosomal proteins and changes in accordance with the distribution of ribosomal proteins as the subunits are associated or dissociated. The proteins should also be associated with the ribosomal particles when they are prepared under a variety of salt and detergent conditions. This criterion is met by the newly assigned proteins under study here. Second, the majority of the protein should be present in fractions containing the ribosome, and there should be little or no free pool of the protein. This criterion is met by the majority of the known MRPs, although there are exceptions, including MRPL12, which is known to have a free pool of protein that may interact with RNA polymerase. Examination of the distribution of the new MRPs classified here indicates that this criterion is also met. Proteins involved in processes such as ribosome assembly generally show a distribution in which only a portion of the total protein is found in the ribosome fraction. For example, less than half of C7orf30, which is involved in the assembly of the large subunit, is observed associated with ribosomes (Rorbach et al., 2012). Similarly, a significant fraction of ERAL1 involved in the assembly of the small subunit is also observed in the non-ribosomal fraction of mitochondria (Dennerlein et al., 2010).

Third, the preparations studied should have few peptide signals from proteins that are known to be transiently associated with the ribosome. In our analysis, slight signals were detected from elongation factor Tu and C7orf30. However, no peptides were detected from many other proteins known to be associated with the ribosome, including elongation factor G, initiation factor 2, initiation factor 3, OXA1L, ObgH1, and Mtg1. Thus, the bulk of the evidence indicates that CHCHD1, CRIF1, and AURKAIPI are, indeed, ribosomal proteins.

With the newly identified MRPs, the number of mammalian mitochondrial ribosomal proteins is brought to 31 for the small subunit and is increased to 51 for the large subunit (Koc et al., 2000, 2001a,b; Suzuki et al., 2001; O'Brien et al., 2005) (Table 4). It is not feasible to assign specific roles for the majority of mitochondrial-specific proteins without structural information and their relative locations in the ribosome; however, many of them have been shown to be essential for mitochondrial protein synthesis and function. For instance, the yeast homologue of MRPS37 (CHCHD1), MRP10, was discovered to be indispensable for mitochondrial translation. A respiratory defect caused by a null mutant of *MRP10* was recovered by the reintroduction of the *MRP10* gene into a wild-type mitochondrial DNA background (Jin et al., 1997). Similarly, MRPS38 (AURKAIP1) is a possible homolog of yeast COX24p, which is involved in the processing and translation of COX I mRNA (Barros et al., 2006). However, we observed an overall reduction in the expression of all 13 mitochondrially encoded proteins in AURKAIP1 knock-down cell lines (Figure 4C), suggesting that this protein plays a general role in mitochondrial translation that is not limited to the synthesis of cytochrome oxidase. Further, no changes in the level of COI mRNA were observed in the AURKAIP1 knock-down cell line (Figure 4A). This observation clearly supports the fact that mammalian mitochondrial genes do not contain introns to be processed, unlike the mitochondrially encoded genes in yeast. MRPS37 and MRPS38 are ribosomal small subunit proteins that are clearly involved in translation; however, their specific roles in mammalian mitochondrial translation remain to be discovered.

The siRNA knock-down of MRPS39 (PTCD3) decreased the activities of Complex III and Complex IV, possibly by directly affecting mRNA binding to the mitochondrial ribosome, as shown in our cross-linking experiments (Haque et al., 2011). In fact, PTCD3 was identified as one of the mRNA-binding proteins and IF3_mt_ interacting proteins located near the mRNA-binding path of the small subunit in crosslinking studies (Davies et al., 2009). Although the majority of the MRPs forming the platform of the mRNA-binding path in the 28S subunit are bacterial homologs (such as MRPS7, MRPS11, MRPS18, and MRPS21), the shoulder region and the mRNA-gate of the 28S subunit are mainly formed by mitochondrial-specific MRPs (Smits et al., 2007; Christian and Spremulli, 2011; Koc and Koc, 2012). Therefore, it is possible that MRPS39 is one of the proteins that forms the mRNA-binding path and interacts with the 5′-ends of mitochondrial mRNAs.

MRPL58 was initially identified as immature colon carcinoma transcript 1 (ICT1) because it was one of the transcripts differentially expressed in colorectal tumors that deviate from the normal maturation pathway in colon epithelium (Van Belzen et al., 1995, 1998). Later, it was discovered to be an unusual member of a release factor family involved in termination of mitochondrial translation, possessing a codon-independent peptidyl-tRNA hydrolase activity associated with the mitochondrial ribosome (Richter et al., 2010). It was also recently reported that MRPL58 is essential for cell viability, because its knock-down resulted in reduced cytochrome *c* oxidase activity and eventually lead to apoptotic cell death (Handa et al., 2010).

The other new large subunit protein, MRPL59 (CRIF1), has been identified as a transcription co-factor that controls the G1/S phase of the cell cycle (Chung et al., 2003). It also negatively regulates the stability of a transcription factor, nuclear respiratory factor 2, that stimulates the expression of many mitochondrial proteins and proteins involved in oxidative damage (Kang et al., 2010). The majority of these studies were performed using the protein tagged at its amino terminus as the ectopically expressed form of CRIF1; therefore, it is possible that the majority of the tagged CRIF1 did not get incorporated into the 55S ribosome, due to the masking of the mitochondrial targeting signal. This form of the expressed protein could subsequently be translocated into the nucleus. Another explanation could be that a very small fraction of CRIF1 is located in the nucleus to manifest its function as a transcription co-factor. However, when the tag is placed at protein's carboxy terminus, this protein is targeted to mitochondria (Chung et al., 2003). Recently, it has been reported to interact with mitochondrial ATAD3 and the ribosome, confirming its mitochondrial localization and assignment as a ribosomal large subunit protein (He et al., 2012; Kim et al., 2012). Endogenous levels of CRIF1 have been shown to be dramatically reduced in epithelial cell cancers in thyroid and breast tissue, in agreement with the possible mitochondrial dysfunction reported in many different cancer types. In this study, we provided very strong evidence for CRIF1 being a *bona fide* ribosomal protein and an essential component of the mitochondrial translation machinery.

Given that the mitochondrial translational machinery and its components are essential for the expression of OXPHOS subunits, studies related to the identification of new components of the translational machinery and their specific roles in translation have the utmost importance in understanding energy production by OXPHOS. A completed picture of the mitochondrial translation machinery will be important to assess mitochondrial dysfunction manifested not only in neurodegenerative diseases, aging, diabetes, and cancer, but also in acute and chronic cardiovascular diseases.

## Funding sources

This work was supported by the National Institutes of Health [R01GM32734 to Linda L. Spremulli, R01GM071034 to Emine C. Koc].

### Conflict of interest statement

The authors declare that the research was conducted in the absence of any commercial or financial relationships that could be construed as a potential conflict of interest.

## Abbreviations

MRPS: Mitochondrial ribosomal small subunit protein
MRPL: Mitochondrial ribosomal large subunit protein
capLC-MS/MS: capillary liquid chromatography-nanoelectrospray ionization-tandem mass spectrometry.
