# Supplementary material for: Identification and characterization of CHCHD1, AURKAIP1, and CRIF1 as new members of the mammalian mitochondrial ribosome
Source: Front Physiol. 2013 Jul 30;4:183. doi: 10.3389/fphys.2013.00183 (PMC3726836; doi:10.3389/fphys.2013.00183)
Supplement: Supplementary file 1 [file Presentation1.PDF]

SUPPLEMENTAL FIGURES AND LEGENDS

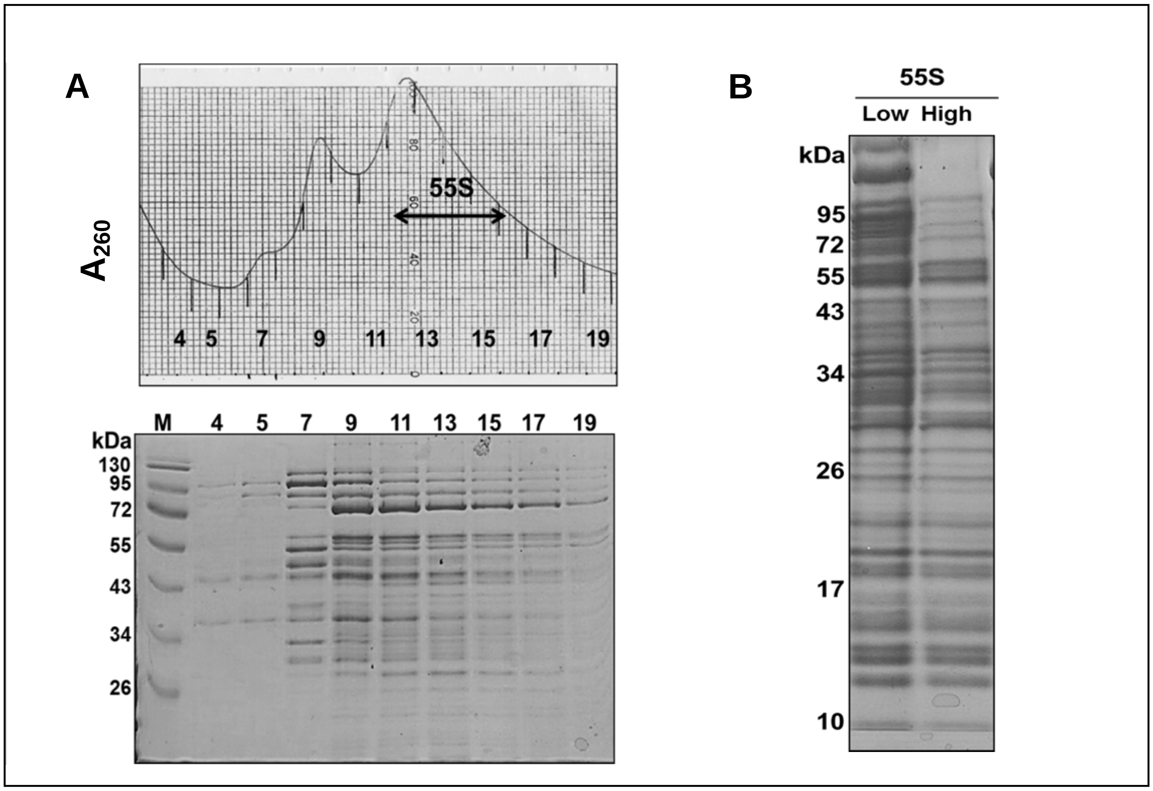

Figure S1. **A.** Approximately 50-60 A_{260} units of high salt and detergent preparations of crude ribosomes were separated on 10-30 % linear sucrose gradient to sediment purified 55S ribosomes (fractions containing purified 55S ribosomes are shown by an arrow). Different subsets of metabolic enzyme complexes were removed by sedimentations performed at high salt and detergent conditions; however, 55S fractions shown by the arrow mainly contained the components of the mitochondrial translation machinery. Sucrose gradient fractions were separated on 12 % SDS-PAGE. **B.** The gradient fractions (shown by the arrow) obtained from high and low salt and detergent preparations were collected as Low and High 55S subunits and 0.5 A_{260} units of each sample were separated on 14 % SDS-PAGE and stained with Coomassie Blue. Mitochondrial ribosomal proteins and ribosome-associated proteins were identified by analyses of in-gel tryptic digests of gel pieces by capLC-MS/MS (Tables S1 and S2). High salt 55S sample was fractionated further for the preparation of purified 28S and 39S subunits shown in Fig. 2A.

Figure S2- Alignment of primary sequences of new mitochondrial ribosomal proteins, MRPS37 (CHCHD1), MRPS38 (AURKAIP1), MRPS39 (PTCD3), MRPL58 (ICT1), and MRPL59 (CRIF1) among different species using ClustalW on Biology Workbench and BOXSHADE. The predicted signal peptide cleavage sites by MITOPROTII are shown by arrows (35).

MRPS37 (CHCHD1)

```
Bovine      1 --MATP-SLRGRLAR-LGNPRKPI-LKPNKPLILANHVGE-RRREKGEATCITEMSIMMA
Human       1 --MATP-SLRGRLAR-FGNPRKPV-LKPNKPLILANRVGE-RRREKGEATCITEMSVMMA
Mouse       1 --MATP-SLRGRLAR-FANPGKPI-LKPNKPLILANRVGN-RRREKGEATCITEMSMMMA
Fly         1 --MRVPGALFAARGR-APQSEKDVPFQEILPLRLKNTVSG-KADSGSDVACLQEMGVLFA
Worm        1 MMFSSPLLKEKALARGKSIYPKVAVFSEILPLASKNRVQAGQKPRAASSSCTQELQALFG
Yeast       1 --------MSGKPPV-YRLPPLPR-LKVKKPII---------RQEAN--KCLVLMSNLLQ

Bovine     55 CWKQNEFRDEACKKEIRDFFDCASR--AEAARKMRSIQED-LGELGS-----LPPRKLNK
Human      55 CWKQNEFRDDACRKEIQGFLDCAAR--AQEARKMRSIQET-LGESGS-----LLPNKLNK
Mouse      55 CWKQNEFRDEACRKEIQDFFDCSSR--AQEARKMRSIQES-LGQSES-----LSPHKMTK
Fly        57 CLKDNEFVEKYCHKEISQFQNCYKCYMDRKFEAKKTVNQG-IVQPGSN----LNYKQLNK
Worm       61 CLKKWEFDDVPCSKQHTLYMDCVHKGAEEAAAYRDATRKGTLGESGAGGKQSMTSAQFNK
Yeast      40 CWSSYGHMSPKCAGLVTELKSCTS---ESALGKRNNVQKSNIN---------YHAARLYD

Bovine    107 LLQRFPNKPHLS----------------------------
Human     107 LLQRFPNKPYLS----------------------------
Mouse     107 LLQRFPNKSHLI----------------------------
Fly       112 YMRRYPNPV-------------------------------
Worm      121 IQKLFPQPDLGKQPYRQMKRLPTQDYADDTFHRKHWSGKRS
Yeast      88 RINGKPHD--------------------------------
```

MRPS38 (AURKAIP1)

```
Bovine          1 MFLMRLTSQLLR-A--VPRAGCSGPWPVLGVLGRHACRPCYSTKPTGPSGVASLPGRRVH
Human           1 MLLGRLTSQLLR-A--VPWAGGRPPWPVSGVLGSRVCGPLYSTSPAGPGRAASLPRKGAQ
Mouse           1 MFLARLTSRLARTV--VPWAGFSRSCPGSGVIGSYAFRPLYSLQPASPSRAASLPGKRTQ
COX24_YEAST     1 -----MLGRALR----PGWLGITR-----TVVKKPSCGSYFN-------------RTFQ
Worm            1 --MSRAVSRLIEACGRLSITQRSQMHFFRPPPTSSILPPLVTPSTTIVEKSIELPSLENP

Bovine         58 MELEEMLVPRKMSISPLESWLTIRYLLPRLDTGAPGTVSPAQLYECPPSRVGEGVEQGGK
Human          58 LELEEMLVPRKMSVSPLESWLTARCFLPRLDTGTAGTVAPPQSYQCPPSQIGEGAEQGDE
Mouse          59 LELEEFLVPRKMAISPLESWLTAQYLLPRRNVEVPVTLAPSQFYECPPRQGEEEAQQGVR
COX24_YEAST    33 TAINTTMPP------MQEGMLSTMMMMTATATRITGTVSEP--------------LNGSN
Worm           59 QQIYTFPTLHRSPIVAPGAILTEILEKLAPTAEKGFLIAPGTPNKSP--------MWLSP

Bovine        118 DVFDAPQMQCRNVLKIRRRKMNHHKYRKLVKRTRFLRRKVREARLKRKQMKFERDLRRIW
Human         118 GVADAPQIQCKNVLKIRRRKMNHHKYRKLVKKTRFLRRKVQEGRLRRKQIKFEKDLRRIW
Mouse         119 EAWDATPVQCKNVLKIRRRKMNHHKYRKLVKRTRFLRRKVREGRLKKKQIKFEKDLKRIW
COX24_YEAST    73 -----IVMQLDSVMRKRKKKMKKHKLRKRRKREKAERRKLSQGR----------------
Worm          111 RLLTIRRKKMKKHKRRRRYDRDFFKYQKYHREKKLKSEREFQKRMKSLLTELEAFNPEKY
Fly*          175 ------KMEAARLIVIRRRKMKKHKLKKLRRKMKFEWAKVRQRREMRKEKAFQAKLISQI

Bovine        178 QKAGLKEAPPGWQTPKIYLKGK--------------------------------------
Human         178 LKAGLKEAPEGWQTPKIYLRGK--------------------------------------
Mouse         179 LKAGLKEAPENWQTPKIYLKNK--------------------------------------
COX24_YEAST       ------------------------------------------------------------
Worm          171 VKDTIRMANKEWQDELAPTGRKLYPHWSRFMSLEQLYGLPKSEYIDKRAGLPTPEEAEQI

Bovine            --------------
Human             --------------
Mouse             --------------
COX24_YEAST       --------------
Worm          231 KALKEKYAKLYRKK
```

(*) Only residues 177-238 were aligned for the Fly homolog (Q8IML6) of AURKAIP1.

MRPS39 (PTCD3)

```
Bovine     1 --------------------------------------------MASVASARWLRVSCGL
Human      1 --------------------------------------------MAVVSAVRWLGLRSRL
Mouse      1 -------------------------------------------MAAAAVAARRLSFRSGL
Fly        1 -------------------------------------------------------MYLSR
Worm       1 ------------------------------------------------------------

Bovine    17 CVPLTARRAGPCGRTPSSRFYSGSAAVPKDEGADIAGTEEVVIPKKKTWDKVAVLQALAS
Human     17 GQPLTGRRAGLCEQARSCRFYSGSATLSKVEGTDVTGIEEVVIPKKKTWDKVAVLQALAS
Mouse     18 VLLQTTRGTGVCEPKVCCRFYAGTESLPKVEGSDITGIEEIVIPKKKTWDKVAVLQALAS
Fly        6 QLRLLPRANIACSLSSSGAHYTTAAPAEDAP---------IEIPNRIERSPTDLLQALAS
Worm       1 ---------MGLGRVITRSLSSVEPALSQK----------LTIQPAIERSPTDLLNALSE

Bovine    77 TVHRDTTAAPYAFQDDPYLIPTSSVESHSFLLAKKSGENAAKFIINSYPKYFQKDIAEPH
Human     77 TVNRDTTAVPYVFQDDPYLMPASSLESRSFLLAKKSGENVAKFIINSYPKYFQKDIAEPH
Mouse     78 TVNRDPTAAPYVFHDDPYLIPTSALESRSFLLAKKSGETAAKFIINSYPKYFQKDIAEPH
Fly       57 TVARDYTAPHYKYHDDPFLIPMSNAAKRTYAMSKESGRKAAKWIKEEHRELFMHQEAQPA
Worm      42 TVGPDTTAPHFAYIDDPITIPSTQSTKKTYFMAKEFGKRAARELATEWPTLFAFDRDQPQ

Bovine   137 IPCLMP-EYFEPQIEEISEAALQERIKLKKVKASVDIFDQLLQAGTTVSLETTNSLLDLL
Human    137 IPCLMP-EYFEPQIKDISEAALKERIELRKVKASVDMFDQLLQAGTTVSLETTNSLLDLL
Mouse    138 IPCLMP-EYFEPQIEDVSEAALEERIRLRKVRASVDMFDQLLQAGTTVSLETTNSLLDLL
Fly      117 IEKFAP-SMVYTEDSVVDETSLAQLISQGELKDAVLVYNLLEQKGNPISPELKQSLLELV
Worm     102 LPVFRPRHLADPLQVEPTENNLLKMISSREVKDSCILYERMRSENVDVSEKVQLELFRLV

Bovine   196 CYYGNQEPSTNYNFQQHEQTEELEEAEEGDNMKSKKKAGHQLGVTWRARNHAERIFALMP
Human    196 CYYGDQEPSTDYHFQQTGQSEALEEE---NDETSRRKAGHQFGVTWRAKNNAERIFSLMP
Mouse    197 CYYGDQEPPADYPFQQTEHLENLEEAAEENNQTSKMESG-----PWKAQNNAERIFALMP
Fly      176 CFHNNQEPIPEEYIEERWFLQ---------NNKRRERSGK----TWKDGDLAEKLYSEIE
Worm     162 TYYNSSNVPFAEWEEFVGMR----------NFGEDSTS------SWKSGAVADLLFETLP

Bovine   256 EKNAHSYCTMIRGMVKH---RAHTQALSMYTELLNNRLRADVHTFNSLIEATALVVNAKF
Human    253 EKNEHSYCTMIRGMVKH---RAYEQALNLYTELLNNRLHADVYTFNALIEATVCAINEKF
Mouse    252 EKNARSYCTMIRGMVKH---RAYAQALNVYTELLNNRLSADVYTFNALIEAKTFILNEKF
Fly      223 PKTPQSYASLIRGMAKY---LQCERAYALLQEAGEKQVQLDTNTFNSVIEIVSFLKDT-A
Worm     206 -KTDETVSIMIAGLCKFSDHSSLERARELYKEHRAAKGKVYREAFNGLIGASSYSVG---

Bovine   313 EEKWNNILDLLKQMVAQNVKPNLQTFNTILKCLRRF--YAFGKLPALQTFREMKAIGIEP
Human    310 EEKWSKILELLRHMVAQKVKPNLQTFNTILKCLRRF--HVFARSPALQVLREMKAIGIEP
Mouse    309 EEKWNDILDLLKHMVAQKVKPNLQTFNTILKGLRKC--YSLGRIPALQILREMKHIGIEP
Fly      279 EQRWQLCKDLLNEMSQQKLRPNLGTLNAVLQCISTFGNFKVARAAALQALPEFKQLGVNP
Worm     262 -------KKLVAEMISLRMTPDIFTFNALLSSAAKAGKFEDRVKAFTEIIGEMKEIGVEP

Bovine   371 SLATYHHIIQ-------LFY-QHESPSKGSSL---------IIYDIMDEITGKTFSPKDP
Human    368 SLATYHHIIR-------LFD-QPGDPLKRSSF---------IIYDIMNELMGKRFSPKDP
Mouse    367 SLATYHHIIH-------LFYPRDLSAIKMPSL---------IIYDIMNELEGRTFSPQDL
Fly      339 SLGSYYYLLI-------IFCRERGP----VSH---------VIVDILNDISGKEFQIQHP
Worm     315 ALSSFHLIIKNIIDFKLLDNEKRESDEQKKTYNHQLTVAISWLNEIQNSLTGKTLKPITS

Bovine   414 DDDMFFQSAMRVCS-SLRDLELAYQVHGLLNTGDNRKFIGPDPRRNFYYSKFFSLLCLME
Human    411 DDDKFFQSAMSICS-SLRDLELAYQVHGLLKTGDNWKFIGPDQHRNFYYSKFFDLICLME
Mouse    411 DDGRFFQLAMSVCS-SLRDLELAYQVHRLLNTGDNRKLVGHDPLRKVYYSKFFSLICSLE
Fly      379 KDTYFFATAMDVCRNHLHDKSLAKKVDELLHTGKNYDLVGDSFKESIYYRNYLALLCQTE
Worm     375 TCNLFFVEAMGIHYRAANENLAENLVSIYESKANEVKMPAFTIESMFYNRQLQLAVEQSA

Bovine   473 QIDVTLKWYKDLIPSVFFPHSQTLIDLLQALDVAN--RLEMIPQIWKD---SKEYGHTFR
Human    470 QIDVTLKWYEDLIPSAYFPHSQTMIHLLQALDVAN--RLEVIPKIWKD---SKEYGHTFR
Mouse    470 QIDVTLKWYKDLIPSVFLPHYQIFIGLLQALDVAN--RLELVPQIWKD---SKEYSHTFR
Fly      439 STEDFMLSYDLLVPNIYIPEPGIMEEILRAIEING--AVEYVPRLWSD---MVVFDHTHR
Worm     435 SLNRIYDLYTTMVPRLVGVNNYLSSLVFRKLAASSDRHWPLLRRVIIDGIAAGQMNGVIG
```

MRPS39 (PTCD3) cont'd

```
Bovine   528 SDLKEEILMLMARDQHPP------ELQAAFADCAADIKSTYESQDARQTASEWPANSLNY
Human    525 SDLREEILMLMARDKHPP------ELQVAFADCAADIKSAYESQPIRQTAQDWPATSLNC
Mouse    525 DALREEVLMLMARDKHPP------ELQVAFADCAADIKSTYEDQSARQPAFDWPANPLQY
Fly      494 ESLLLYVLRILVDNKPNPDSPAQAQLPEQGAKVALDMFERVEEAIKRLRKVSFTGQMLGD
Worm     495 EEMRKQLCNVQLHTLGTSEREQFTSLVQKLVAVWVEFSQFTEERMRRLQRKLSPSQ-ISE

Bovine   582 IAILFLRAGRTQEAWKMLGLFRKHNK-------------IPRNELLNEFMDSAKASSSPA
Human    579 IAILFLRAGRTQEAWKMLGLFRKHNK-------------IPRSELLNELMDSAKVSNSPS
Mouse    579 IAVLFLRGGRSQEAWKMLELFKKHKK-------------IPRNELLEEFMDTAKASGSTA
Fly      554 ILTLLVRGGSYEKATEVFAHIDKNQHRIPG---------TPSLNALIEFVDASVQEKSPS
Worm     554 CALLLTRIGEQQKAYELLDLLLDETASSGDEATVYPRGHARPWAMAELFEDALRKRDTYA

Bovine   629 QAVEVVKLANSFSLPICEGLTQRLTADFTLSQEQKEALGDLTALTSDSE----SDSDSDT
Human    626 QAIEVVELASAFSLPICEGLTQRVMSDFAINQEQKEALSNLTALTSDSDTDSSSDSDSDT
Mouse    626 LAIEVVKLASAFSLPIGESLAQRVVMDFTVDPEQKEALGNLTELNSSDG---ESSSDSDS
Fly      605 QALFALQYAVENNFDSRE-LAKRIHEGFTLNETHLSKLKSLVGESFLDK-----------
Worm     614 AATCLQIMSLTANRAKLEPLANRIFEKCNVNQEQVRIIQGFIRLRPQ-------------

Bovine   685 SKDK
Dog      728 SEGI
Human    686 SEGK
Mouse    683 DDK-
```

MRPL58 (ICT1)

```
Mouse     1 -----MATAWGLRWGLSRTGTLLLAPPARCARRALHRQEH--------------------
Human     1 -----MAATRCLRWGLSRAGVWLLPPPARCPRRALHKQKDGTEFKSIYSLDKLYPESQGS
Bovine    1 -----MAAARCLRWGLSRAEAWLLPPPTSCCHRALHRQVEGTEFRSAYSLDKLYPESRGA
Fly       1 MNKITSAFIRVLRQSSSSGGTGNLLGRQLSYKSDLSLDKIYPG------------ARLQI
Worm      1 -----------MLRNLSS----------FVVKSTRHIQAS--------------------

Mouse    36 ---------AKQASSYIPLDRLSISYCRSSGPGGQNVNKVNSKAEVRFHLASADWIEEPV
Human    56 DTAWRVPNGAKQADSDIPLDRLTISYCRSSGPGGQNVNKVNSKAEVRFHLATAEWIAEPV
Bovine   56 DTAWRVPGDAKQGNDDIPVDRLTISYCRSSGPGGQNVNKVNSKAEVRFHLASADWIAEPV
Fly      49 YTPPPPPSGSDKFSGFIPMDRLEITYSRSSGPGGQHVNTVNTKVDVRFKVAQADWIPEQT
Worm     20 --------SSATFNGVIPTEKIEKRYTLSSGPGGQNVQKNATKVEIRFKVSEAEWLSESL

Mouse    87 RQKIALTHKNKINKAGELVLTSESSRYQFRNLAECLQKIRDMI-AEASQVPK-EPSKEDA
Human   116 RQKIAITHKNKINRLGELILTSESSRYQFRNLADCLQKIRDMI-TEASQTPK-EPTKEDV
Bovine  116 RQKMALTHKNKINRAGELILTSEYSRYQFRNLADCLQKIRDMI-AEASQPAT-EPSKEDA
Fly     109 RQKLLKVLANRITKDGYFYIKSDLTRSQQMNLADALEKLRTIIRSQEAVVPA-PPSEETL
Worm     72 RDLVEEKLSHRINTAGELIIDSDRTRERHLNVADCFDKLRSAIYAIENEQGKREMTEKDE

Mouse   145 RLQRLRIEKMNRERLRQKRLNSALKTSRR--MTMD-
Human   174 KLHRIRIENMNRERLRQKRIHSAVKTSRR--VDMD-
Bovine  174 ALQKLRIENMNRERLRKKRINSAIKTSRR--VGTD-
Fly     168 EKLRRRQERAVRERLQLKRGRAQVKADRQGPSGLDL
Worm    132 KILRERAAIATQHRLQEKRRTSEKKASRRAAVEF--
```

MRPL59 (CRIF1)

```
Bovine     1 MAAPVRQTRSLLGWVTTLGPGSRGYRAPPPPRRS----REPWWPDPDDP--LTPRWQ---
Human      1 MAASVRQARSLLGVAATLAPGSRGYRARPPPRRR----PGPRWPDPEDL--LTPRWQ---
Mouse      1 MAALAMRSGYLLRLSVALGPRSRSYRAPPPPRRR----PGPHSPDPENL--LTPRWQ---
Fly        1 MNVGKISVVTRLPALRSCAQYSSAAKAELPASLVGDVDVEPTYPQTVDRSGLQPQHKNVL
Worm       1 MLRNWRLLVRSFATNSSEEIAPKVDVSFLRPRHR------IIAAGGMPPVQFDSERE---

Bovine    52 --------------LGPRYAAKQFARHGAASGVDPGSLWPSREQLLELEAEEREWYPSLA
Human     52 --------------LGPRYAAKQFARYGAASGVVPGSLWPSPEQLRELEAEEREWYPSLA
Mouse     52 --------------LTPRYVAKQFGRHGAISGVPPASLWPTPEQLRELEAEEQEWYPSLA
Fly       61 LNKLPYQEPHSWIHLTEKYQRQAFGRYGAQSNVNPKICFDSHG---EKDSRQVMQLETLL
Worm      52 ----------------RASRRERFGKYGLKSGVPVEELFPTAEEIEEEEAIG--LFREFN

Bovine    98 VMQESLRVQQLAEEQKRQAREQLIEECMAKMPQMIENWRRQQQARREKAQADKERRARLQ
Human     98 TMQESLRVKQLAEEQKRREREQHIAECMAKMPQMIVNWQQQQRENWEKAQADKERRARLQ
Mouse     98 TMQESLRLQQQALEARRQAREQRIAECMAKMPQMIENWRKQKREREWEKIQADKERRARLQ
Fly      118 KMLEKNRAQKAEELARINAREEDIAKKMEKLTQWKADLHAKIAKREADAAAAIQRKERLV
Worm      94 NAKKEYKELQKKKRESELARIAELEKNLKKYPATLAKYEASLVKQEQEKDEKEVALEKRI

Bovine   158 AEAQERLGYHVDPRSARFQELLQDLEKQHRKRLKEEKQRKKKEARAAAMAAAAAQDPADS
Human    158 AEAQELLGYQVDPRSARFQELLQDLEKKERKRLKEEKQKRKKEARAAALAAAVAQDPAAS
Mouse    158 AEAQERLGYHVDPRSARFQELLQDLDKQQRKRLKEERQRQKKEARIAAMASAEAQDSAVS
Fly      178 EEVRRHFGFKVDTRDERFKEMLEQKEKEDKKKQKEAKRKAKEEKMMAKLVEKASV-----
Worm     154 RDIQEYFGYWMDPKDPRFEVMLQQKEAEEKKAVKMAKRDEIQKKRFAENVQG--------

Bovine   218 ETPDS
Human    218 GAPSS
Mouse    218 GEPSS
Fly          -----
Worm         -----
```
