# Supplementary material for: Identification and characterization of CHCHD1, AURKAIP1, and CRIF1 as new members of the mammalian mitochondrial ribosome
Source: Front Physiol. 2013 Jul 30;4:183. doi: 10.3389/fphys.2013.00183 (PMC3726836; doi:10.3389/fphys.2013.00183)
Supplement: Supplementary file 2 [file DataSheet1.PDF]

Table S1. Bovine mitochondrial ribosomal proteins identified in the LC-MS/MS analyses of 28S, 39S, and 55S samples prepared at high salt and detergent conditions and their total Mascot scores.

	Mascot Scores					
**Protein**	**28S**	**39S**	**55S**	**Fractions***	**MW (Da)**	**Acc. Number**
MRPS2	309	101	176	14	31672	P82923
MRPS5	821	99	501	10, 10, 9	47842	Q2KID9
MRPS6	199	117	86	28, 27, 26	14139	P82931
MRPS7	743	258	454	17, 16, 16	28112	Q3T040
MRPS9	2014	124	557	10, 10, 9	45202	Q58DQ5
MRPS10	911	117	236	21, 22, 23	23017	P82670
MRPS11	733	293	358	23, 21, 21	20809	F1N498
MRPS12	122	ND	74	27, 25	15437	Q29RU1
MRPS14	854	235	386	27, 26, 25	15034	Q6B860
MRPS15	564	69	206	14, 17, 16	29172	E1BBB4
MRPS16	644	98	140	26, 25, 25	15122	P82915
MRPS17	343	64	124	24, 23, 22	14437	E1BF33
MRPS18A	ND	121	89	21	22219	F1MJC2
MRPS18B	437	83	288	13, 14	29192	F1N059
MRPS18C	190	ND	66	25, 24	16203	P82917
MRPS21	802	179	269	27, 30, 30	10668	P82920
MRPS22	1786	ND	966	10, 20	40653	P82649
MRPS23	794	265	328	19, 18	21564	F1MZW1
MRPS24	368	123	114	23, 25	19067	Q2M2T7
MRPS25	1090	132	390	22, 23, 24	20042	P82669
MRPS26	540	238	249	16, 20, 22	23759	Q3SZ86
MRPS27	1479	205	742	10, 10, 8	47891	F1N1S1
MRPS28	225	117	126	21, 26	21028	P82928
MRPS29	1645	362	896	8, 10	48413	P82922
MRPS30	288	581	851	7, 8	49325	P82924
MRPS31	134	67	76	9, 13, 19	43648	P82925
MRPS34	673	ND	389	16, 15	25843	F1MKN3
MRPS33	211	66	88	24, 28	12445	P82926
MRPS35	1524	282	732	9, 11	37046	Q2YDF6
MRPS36	375	228	162	27, 29	11536	P82908
MRPS37 (CHCHD1)	810	ND	680	27, 25	13546	Q2HJE8
MRPS38 (AURKAIP1)	263	175	238	23, 24, 23	22906	Q0VCJ1
MRPS39 (PTCD3)	2394	409	1816	4, 4, 3	77781	Q2KI62
MRPL1	350	415	506	13, 14, 12	36633	A6QPQ5
MRPL2	ND	92	440	18, 14	33323	Q2TA12
MRPL3	207	194	278	12, 12, 10	38575	Q3ZBX6
MRPL4	435	336	347	13, 13, 11	33350	Q32PI6
MRPL9	333	498	542	18, 16, 16	30506	Q2TBK2
MRPL10	ND	134	153	16, 15	29309	Q3MHY7
MRPL11	557	777	599	19, 19, 19	20767	Q2YDI0
MRPL12	327	438	311	21, 20, 19	21387	Q7YR75
MRPL13	305	400	302	21, 20, 20	20562	Q3SYS1

MRPL14	172	308	247	27, 26, 25	15905	Q1JQ99
MRPL15	1003	740	1163	13, 14, 12	33653	Q0VC21
MRPL16	101	208	142	17, 16, 16	28555	Q3T0J3
MRPL17	294	178	360	23, 22, 22	19733	Q3T0L3
MRPL18	297	493	538	23, 22, 22	20384	Q3ZBR7
MRPL19	395	179	431	14, 14, 13	33284	F1MMM8
MRPL20	169	269	242	21, 21, 21	17524	Q2TBR2
MRPL21	193	230	205	21, 25	23135	F1MSV8
MRPL22	565	635	657	22, 21, 21	23537	Q3SZX5
MRPL23	ND	ND	78	22	12307	Q32PA7
MRPL24	478	345	395	17, 16, 15	24770	Q3SYS0
MRPL27	415	400	338	28, 26, 26	16024	Q32PC3
MRPL28	ND	84	101	12, 14	30021	Q2HJJ1
MRPL30	159	162	160	25, 23, 23	18486	Q58DV5
MRPL32	ND	54	149	24, 25	21310	Q2TBI6
MRPL33	251	352	97	30, 30, 28	7485	Q3SZ47
MRPL34	183	178	111	30, 30, 30	10747	A8NN94
MRPL35	ND	53	ND	24	21637	Q3SZA9
MRPL36	ND	ND	ND	-	10750	XP_580819
MRPL37	719	822	1363	10, 10, 8	48022	A4FUC0
MRPL38	353	254	380	10, 10, 9	44594	Q3ZBF3
MRPL39	376	312	499	1, 10, 9	38435	Q05B74
MRPL40	ND	ND	216	20	23690	F1N1R3
MRPL41	254	258	270	27, 26, 25	15012	A5PJ71
MRPL42	238	170	69	27, 25, 25	16626	P82927
MRPL43	513	658	566	24, 23, 23	17724	Q95KE5
MRPL44	521	603	579	13, 13, 11	37389	Q2KIS2
MRPL45	209	185	230	13, 14, 12	35120	Q3T142
MRPL46	493	235	487	16, 14, 14	31710	F1N499
MRPL47	342	629	500	19, 18, 18	29562	Q08DT6
MRPL48	455	ND	417	16, 19	24088	F1MEK4
MRPL49	370	396	341	26, 25, 24	19235	F1MQ25
MRPL50	358	237	356	27, 25, 25	18055	Q2KI49
MRPL51	172	124	151	28, 26, 27	15152	P0C2B6
MRPL52	ND	71	ND	26	13804	P0C2B7
MRPL53	ND	250	187	27, 26	11990	Q2HJF1
MRPL54	ND	68	64	27	16307	E1B8J3
MRPL55	ND	110	95	26	14999	P0C2B8
MRPL56 (LACTB)	820	845	737	6, 7, 5	61614	A5PJ81
MRPL57 (MRP63)	135	258	102	30, 27, 26	11859	Q3ZC04
MRPL58 (ICT1)	436	433	473	21, 21, 20	23292	Q3T116
MRPL59 (CRIF1)	219	435	613	19, 18, 17	25730	A1A4P4
mtEF-Tu	ND	ND	163	7	49367	P49410
C7orf30	110	183	116	21, 21, 20	26087	Q0P562

ND; Not detected.

(*) Gel fraction numbers (Fig. 3A) corresponding to the highest Mascot scores listed for each MRP found in 28S, 39S, and 55S samples.

Table S2. Common contaminant proteins identified in the LC-MS/MS analysis of in-gel tryptic peptides of 28, 39S, and 55S samples prepared at high salt and detergent conditions.

SwissProt/ Uniprot Number	MW (Da)	Name	28S	39S	55S
F1ML89	164636	Carbamoyl phosphate synthase 1 (CPS1)	✗	✓	✓
Q148N0	115734	2-oxoglutarate dehydrogenase (OGDH)	✓	✓	✓
E1BFI2	103041	2-oxoglutarate dehydrogenase E1 component	✓	✗	✓
O62828	84762	Mitochondrial trifunctional protein subunit alpha (HADHA)	✓	✓	✓
F6QNC4	81616	Propionyl-CoA carboxylase alpha chain (PCCA)	✓	✗	✗
E1BGC1	80432	Methylcrotonoyl-CoA carboxylase subunit (MCCC1)	✓	✗	✗
F1ML97	77288	Nitric oxide synthase 1 C4orf14 (C6H4ORF14)	✓	✗	✗
Q3ZCH0	73696	Stress-70 protein HSPA9	✓	✓	✓
F1MDS3	70605	Phosphoenolpyruvate carboxykinase	✗	✗	✓
F1N690	69023	dihydrolipoyllysine-residue acetyltransferase activity (DLAT)	✓	✓	✓
P00366	61473	Glutamate dehydrogenase 1 (GLUD1)	✓	✗	✗
E1BPP6	61253	Methylcrotonoyl-CoA carboxylase beta (MCCC2)	✓	✓	✗
F1MUZ9	60939	60 kDa heat shock protein (HSPD1)	✓	✗	✗
P19483	59683	ATP synthase subunit alpha (ATP5A1)	✓	✓	✓
Q2TBR0	58274	Propionyl-CoA carboxylase beta chain (PCCB)	✓	✓	✗
P20000	56617	Aldehyde dehydrogenase (ALDH2)	✓	✓	✓
P00829	56249	ATP synthase subunit beta (ATP5B)	✓	✓	✓
F1N206	54153	Dihydrolipoyl dehydrogenase (DLD)	✓	✓	✓
P22439	53852	Pyruvate dehydrogenase protein X component (PDHX)	✓	✓	✓
A5D9E7	53670	Mitochondrial trifunctional protein, beta subunit (HADHB)	✓	✓	✓
P11181	53376	Lipoamide acyltransferase component of branched-chain alpha-keto acid dehydrogenase complex (DBT)	✓	✓	✓
P11178	51702	2-oxoisovalerate dehydrogenase subunit alpha (BCKDHA)	✓	✓	✓
P11179	48942	Dihydrolipoyllysine-residue succinyltransferase component of 2-oxoglutarate dehydrogenase complex (DLST)	✓	✓	✓
P23004	48119	Cytochrome b-c1 complex subunit 2 (UQCRC2)	✗	✗	✓

Q2KJG8	46409	3-methyl-2-oxobutanoate dehydrogenase lipoamide kinase (BCKDK)	✗	✓	✗
Q29RZ0	44860	Acetyl-CoA acetyltransferase (ACAT1)	✓	✗	✗
A7MB35	43360	Pyruvate dehydrogenase E1 component subunit alpha (PDHA)	✓	✓	✓
Q2T9Y3	43264	Pyruvate dehydrogenase (Lipoamide) alpha 2 (PDHA2)	✗	✗	✓
P21839	42908	2-oxoisovalerate dehydrogenase subunit beta (BCKDHB)	✓	✗	✗
E1BPG0	42197	malonyl CoA:ACP acyltransferase (MCAT)	✓	✗	✗
Q3T0R7	42105	3-ketoacyl-CoA thiolase (ACAA2)	✓	✗	✗
P11966	39101	Pyruvate dehydrogenase E1 component subunit beta (PDHB)	✓	✓	✓
Q1JPJ6	38870	Pyruvate dehydrogenase kinase, isoenzyme 2 (PDK2)	✗	✗	✓
Q58DM8	31223	Enoyl-CoA hydratase (ECHS1)	✓	✓	✗
O02691	27123	3-hydroxyacyl-CoA dehydrogenase type-2 (HSD17B10)	✓	✗	✗
F1N1S0	17181	Single-stranded DNA-binding protein (SSBP1)	✓	✗	✗

✓; Detected
✗; Not detected

Table S3. Peptides with unique parent ions (m/z) detected by LC-MS/MS analyses of 28, 39S, and 55S samples used in emPAI calculations of new MRPs.

Peptide Seq.	Score	m/z	Exp. Mr	Calc. Mr	Pep. Δ
**CHCHD1 peptides in 28S**					
KPILKPNKPLILANHVGER	71.92	713.9359	2138.7859	2136.2895	2.4964
KPILKPNKPLILANHVGER	91.09	713.2415	2136.7027	2136.2895	0.4132
KPILKPNKPLILANHVGER	89.99	713.8925	2138.6557	2136.2895	2.3662
KPILKPNKPLILANHVGER	87.97	713.9501	2138.8285	2136.2895	2.5391
SIQEDLGELGSLPPR	61.17	805.8832	1609.7518	1609.8311	-0.0793
SIQEDLGELGSLPPR	92.49	805.9407	1609.8668	1609.8311	0.0357
SIQEDLGELGSLPPR	88.58	806.1552	1610.2958	1609.8311	0.4647
SIQEDLGELGSLPPR	89	806.1857	1610.3568	1609.8311	0.5257
SIQEDLGELGSLPPR	92.14	806.3132	1610.6118	1609.8311	0.7807
SIQEDLGELGSLPPR	112.43	805.7277	1609.4408	1609.8311	-0.3903
SIQEDLGELGSLPPR	88.76	806.1741	1610.3336	1609.8311	0.5025
SIQEDLGELGSLPPR	106.38	806.1917	1610.3688	1609.8311	0.5377
SIQEDLGELGSLPPR	102.68	806.2227	1610.4308	1609.8311	0.5997
SIQEDLGELGSLPPR	108.77	806.2372	1610.4598	1609.8311	0.6287
SIQEDLGELGSLPPR	112.88	806.2512	1610.4878	1609.8311	0.6567
SIQEDLGELGSLPPR	102.94	806.4136	1610.8126	1609.8311	0.9815
SIQEDLGELGSLPPR	97.34	806.6122	1611.2098	1609.8311	1.3787
SIQEDLGELGSLPPR	82.54	806.1542	1610.2938	1609.8311	0.4627
SIQEDLGELGSLPPR	80.74	806.1587	1610.3028	1609.8311	0.4717
SIQEDLGELGSLPPR	103.91	806.1682	1610.3218	1609.8311	0.4907
SIQEDLGELGSLPPR	101.49	806.2072	1610.3998	1609.8311	0.5687
SIQEDLGELGSLPPR	94.9	806.2177	1610.4208	1609.8311	0.5897
SIQEDLGELGSLPPR	102.46	806.2361	1610.4576	1609.8311	0.6265
SIQEDLGELGSLPPR	79.15	806.6761	1611.3376	1609.8311	1.5065
SIQEDLGELGSLPPRK	63.29	870.2007	1738.3868	1737.9261	0.4608
SIQEDLGELGSLPPRK	71.01	870.2867	1738.5588	1737.9261	0.6328
SIQEDLGELGSLPPRK	81.61	870.3586	1738.7026	1737.9261	0.7766
SIQEDLGELGSLPPRK	72.71	870.7147	1739.4148	1737.9261	1.4888
SIQEDLGELGSLPPRK	57.49	870.7272	1739.4398	1737.9261	1.5138
SIQEDLGELGSLPPRK	50.23	580.9835	1739.9287	1737.9261	2.0026
SIQEDLGELGSLPPRK	80.49	581.2018	1740.5836	1737.9261	2.6575
SIQEDLGELGSLPPRK	49.13	581.2208	1740.6406	1737.9261	2.7145
**CHCHD1 peptides in 55S**					
KPILKPNKPLILANHVGER	79.8	712.2648	2133.7726	2136.2895	-2.5169
KPILKPNKPLILANHVGER	118.68	1069.573	2137.1314	2136.2895	0.842
KPILKPNKPLILANHVGER	75.89	713.6085	2137.8037	2136.2895	1.5142
KPILKPNKPLILANHVGER	78.72	713.8452	2138.5138	2136.2895	2.2243
KPILKPNKPLILANHVGER	118.36	1069.54	2137.0654	2136.2895	0.776
KPILKPNKPLILANHVGER	74.2	713.5005	2137.4797	2136.2895	1.1902
KPILKPNKPLILANHVGER	86.16	713.7569	2138.2489	2136.2895	1.9594
SIQEDLGELGSLPPR	96.45	806.1741	1610.3336	1609.8311	0.5025
SIQEDLGELGSLPPR	98.7	806.2507	1610.4868	1609.8311	0.6557
SIQEDLGELGSLPPR	90.36	806.6837	1611.3528	1609.8311	1.5217

Peptide Seq.	Score	m/z	Exp. Mr	Calc. Mr	Pep. Δ
**CHCHD1 peptides in 55S cont'd.**					
SIQEDLGELGSLPPR	50.97	538.3572	1612.0498	1609.8311	2.2186
SIQEDLGELGSLPPR	95.53	806.2197	1610.4248	1609.8311	0.5937
SIQEDLGELGSLPPR	100.64	806.2336	1610.4526	1609.8311	0.6215
SIQEDLGELGSLPPR	106.65	806.2886	1610.5626	1609.8311	0.7315
SIQEDLGELGSLPPR	81.27	806.3827	1610.7508	1609.8311	0.9197
SIQEDLGELGSLPPR	53.01	806.5312	1611.0478	1609.8311	1.2167
SIQEDLGELGSLPPR	59.08	806.6367	1611.2588	1609.8311	1.4277
SIQEDLGELGSLPPR	50.41	806.8367	1611.6588	1609.8311	1.8277
SIQEDLGELGSLPPR	78.53	806.8522	1611.6898	1609.8311	1.8587
SIQEDLGELGSLPPR	49.64	807.2717	1612.5288	1609.8311	2.6977
SIQEDLGELGSLPPR	87.99	805.9987	1609.9828	1609.8311	0.1517
SIQEDLGELGSLPPR	95.47	806.6772	1611.3398	1609.8311	1.5087
SIQEDLGELGSLPPR	98.86	806.7936	1611.5726	1609.8311	1.7415
SIQEDLGELGSLPPR	63.66	805.9236	1609.8326	1609.8311	0.0015
SIQEDLGELGSLPPR	88.94	806.1506	1610.2866	1609.8311	0.4555
SIQEDLGELGSLPPR	77.37	806.4327	1610.8508	1609.8311	1.0197
SIQEDLGELGSLPPRK	58.05	870.3997	1738.7848	1737.9261	0.8588
SIQEDLGELGSLPPRK	74.5	870.0146	1738.0146	1737.9261	0.0886
SIQEDLGELGSLPPRK	58.32	870.8597	1739.7048	1737.9261	1.7788
**AURKAIP1 peptides in 28S**					
AGLKEAPPGWQTPK	65.1	740.7422	1479.4698	1478.7881	0.6817
AGLKEAPPGWQTPK	70.78	740.9052	1479.7958	1478.7881	1.0077
EAPPGWQTPK	45.66	556.5251	1111.0356	1109.5506	1.4851
EAPPGWQTPK	56.55	556.5927	1111.1708	1109.5506	1.6203
EAPPGWQTPK	55.3	557.1462	1112.2778	1109.5506	2.7273
EAPPGWQTPK	54.08	556.6182	1111.2218	1109.5506	1.6713
**AURKAIP1 peptides in 39S**					
AGLKEAPPGWQTPK	69.9	740.6952	1479.3758	1478.7881	0.5877
AGLKEAPPGWQTPK	68.36	494.4712	1480.3918	1478.7881	1.6036
EAPPGWQTPK	52.12	555.2527	1108.4908	1109.5506	-1.0597
EAPPGWQTPK	50.32	556.1351	1110.2556	1109.5506	0.7051
EAPPGWQTPK	62.77	556.0491	1110.0836	1109.5506	0.5331
**AURKAIP1 peptides in 55S**					
AGLKEAPPGWQTPK	74.27	740.7236	1479.4326	1478.7881	0.6445
AGLKEAPPGWQTPK	60.88	494.3905	1480.1497	1478.7881	1.3615
AGLKEAPPGWQTPK	69.11	740.8422	1479.6698	1478.7881	0.8817
AGLKEAPPGWQTPK	51.52	494.3889	1480.1449	1478.7881	1.3567
EAPPGWQTPK	59.81	555.5881	1109.1616	1109.5506	-0.3889
EAPPGWQTPK	82.7	555.6782	1109.3418	1109.5506	-0.2087
EAPPGWQTPK	55.29	555.8317	1109.6488	1109.5506	0.0983
**PTCD3 peptides in 28S**					
DEGADIAGTEEVVIPK	71.51	821.9131	1641.8116	1641.8097	0.0019
DEGADIAGTEEVVIPK	108.8	821.9807	1641.9468	1641.8097	0.1371

Peptide Seq.	Score	m/z	Exp. Mr	Calc. Mr	Pep. Δ
**PTCD3 peptides in 28S cont'd.**					
VAVLQALASTVHR	82.25	682.8851	1363.7556	1363.7936	-0.0379
VAVLQALASTVHR	75.37	683.2432	1364.4718	1363.7936	0.6783
VAVLQALASTVHR	78.71	683.4227	1364.8308	1363.7936	1.0373
AGHQLGVTWR	54.93	562.8462	1123.6778	1123.5887	0.0892
AGHQLGVTWR	56.5	563.3682	1124.7218	1123.5887	1.1332
AGHQLGVTWR	48.91	564.1061	1126.1976	1123.5887	2.609
AHTQALSMYTELLNNR	48.75	621.3369	1860.9889	1860.9152	0.0737
AHTQALSMYTELLNNR	143.19	931.5956	1861.1766	1860.9152	0.2615
AHTQALSMYTELLNNR	112.19	932.3387	1862.6628	1860.9152	1.7477
AHTQALSMYTELLNNR	85.98	932.8422	1863.6698	1860.9152	2.7547
AHTQALSMYTELLNNR	110	939.8561	1877.6976	1876.9101	0.7875
AHTQALSMYTELLNNR	52.21	627.4459	1879.3159	1876.9101	2.4058
ADVHTFNSLIEATALVVNAK	65.84	705.5242	2113.5508	2112.1215	1.4293
ADVHTFNSLIEATALVVNAK	141.04	1057.836	2113.6574	2112.1215	1.5359
ADVHTFNSLIEATALVVNAK	101	1057.888	2113.7614	2112.1215	1.6399
ADVHTFNSLIEATALVVNAK	112.69	1057.9	2113.7854	2112.1215	1.6639
ADVHTFNSLIEATALVVNAK	71.36	705.7925	2114.3557	2112.1215	2.2342
WNNILDLLK	49.45	564.7062	1127.3978	1127.6339	-0.236
WNNILDLLK	53.63	564.9672	1127.9198	1127.6339	0.286
WNNILDLLK	54.45	564.9852	1127.9558	1127.6339	0.322
WNNILDLLK	49.7	565.1747	1128.3348	1127.6339	0.701
WNNILDLLK	48.74	566.2101	1130.4056	1127.6339	2.7718
QMVAQNVKPNLQTFNTILK	103.26	1094.5	2186.9854	2186.1881	0.7973
QMVAQNVKPNLQTFNTILK	73.77	1094.535	2187.0554	2186.1881	0.8673
QMVAQNVKPNLQTFNTILK	78.37	730.2189	2187.6349	2186.1881	1.4467
QMVAQNVKPNLQTFNTILK	96.57	1102.503	2202.9914	2202.1831	0.8084
GSSLIIYDIMDEITGK	117.77	878.3242	1754.6338	1753.8808	0.7531
GSSLIIYDIMDEITGK	95.67	885.8202	1769.6258	1769.8757	-0.2499
GSSLIIYDIMDEITGK	91.21	886.1757	1770.3368	1769.8757	0.4611
GSSLIIYDIMDEITGK	97.72	886.8452	1771.6758	1769.8757	1.8001
GSSLIIYDIMDEITGK	102.42	887.1727	1772.3308	1769.8757	2.4551
TFSPKDPDDDMFFQSAMR	59.01	717.9432	2150.8078	2149.9085	0.8993
TFSPKDPDDDMFFQSAMR	71.07	723.6356	2167.885	2165.9034	1.9815
DPDDDMFFQSAMR	91.85	795.1307	1588.2468	1589.6127	-1.3658
DPDDDMFFQSAMR	83.2	795.9111	1589.8076	1589.6127	0.195
DPDDDMFFQSAMR	79.59	796.2527	1590.4908	1589.6127	0.8782
DPDDDMFFQSAMR	91.55	804.2437	1606.4728	1605.6076	0.8653
DLELAYQVHGLLNTGDNR	76.28	676.9085	2027.7037	2027.0072	0.6965
DLELAYQVHGLLNTGDNR	109.72	1014.867	2027.7194	2027.0072	0.7122
DLELAYQVHGLLNTGDNR	115.45	1014.939	2027.8634	2027.0072	0.8562
DLELAYQVHGLLNTGDNR	73.1	677.5255	2029.5547	2027.0072	2.5475
DLELAYQVHGLLNTGDNRK	74.41	719.3339	2154.9799	2155.1022	-0.1223
DLELAYQVHGLLNTGDNRK	98.75	1078.976	2155.9374	2155.1022	0.8353
DLELAYQVHGLLNTGDNRK	73.01	720.1312	2157.3718	2155.1022	2.2696
LEMIPQIWK	54.72	579.3832	1156.7518	1156.6314	0.1204
LEMIPQIWK	48.66	579.9692	1157.9238	1156.6314	1.2924
LEMIPQIWK	45.17	587.7787	1173.5428	1172.6263	0.9165

Peptide Seq.	Score	m/z	Exp. Mr	Calc. Mr	Pep. Δ
**PTCD3 peptides in 28S cont'd.**					
SDLKEEILMLMAR	84.96	774.8137	1547.6128	1547.8051	-0.1922
SDLKEEILMLMAR	90.37	775.4371	1548.8596	1547.8051	1.0546
SDLKEEILMLMAR	73.35	782.8337	1563.6528	1563.8	-0.1472
SDLKEEILMLMAR	74.55	783.3217	1564.6288	1563.8	0.8288
SDLKEEILMLMAR	61.59	783.8846	1565.7546	1563.8	1.9546
SDLKEEILMLMAR	57.64	791.0327	1580.0508	1579.7949	0.2559
SDLKEEILMLMAR	51.72	791.5562	1581.0978	1579.7949	1.3029
NELLNEFMDSAK	73.23	706.4631	1410.9116	1409.6496	1.262
NELLNEFMDSAK	48.37	706.7612	1411.5078	1409.6496	1.8582
NELLNEFMDSAK	72.97	713.7452	1425.4758	1425.6446	-0.1687
NELLNEFMDSAK	64.93	714.2567	1426.4988	1425.6446	0.8543
NELLNEFMDSAK	50.78	714.7767	1427.5388	1425.6446	1.8943
ASSSPAQAVEVVK	73.69	637.3401	1272.6656	1271.6721	0.9935
ASSSPAQAVEVVK	64.77	637.9052	1273.7958	1271.6721	2.1237
LTADFTLSQEQK	77.06	691.2942	1380.5738	1379.6933	0.8806
LTADFTLSQEQK	82.44	691.4106	1380.8066	1379.6933	1.1134
EALGDLTALTSDSESDSDSDTSKDK	87.18	863.8135	2588.4187	2586.1304	2.2883
**PTCD3 Peptides in 39S**					
DEGADIAGTEEVVIPK	110.93	821.7332	1641.4518	1641.8097	-0.3579
VAVLQALASTVHR	88.15	683.0427	1364.0708	1363.7936	0.2773
AGHQLGVTWR	51.96	562.6902	1123.3658	1123.5887	-0.2228
AGHQLGVTWR	52.71	563.5582	1125.1018	1123.5887	1.5132
AHTQALSMYTELLNNR	103.1	939.5972	1877.1798	1876.9101	0.2697
LEMIPQIWK	53.02	587.8922	1173.7698	1172.6263	1.1435
NELLNEFMDSAK	74.02	706.1161	1410.2176	1409.6496	0.568
NELLNEFMDSAK	65.62	714.0641	1426.1136	1425.6446	0.4691
ASSSPAQAVEVVK	66.19	636.9617	1271.9088	1271.6721	0.2367
LTADFTLSQEQK	95.23	691.0052	1379.9958	1379.6933	0.3026
LTADFTLSQEQK	72.03	692.2587	1382.5028	1379.6933	2.8096
**PTCD3 Peptides in 55S**					
DEGADIAGTEEVVIPK	106.79	822.2787	1642.5428	1641.8097	0.7331
TWDKVAVLQALASTVHR	141.64	948.4471	1894.8796	1894.0425	0.8372
TWDKVAVLQALASTVHR	70.38	632.7319	1895.1739	1894.0425	1.1314
VAVLQALASTVHR	75.47	682.9501	1363.8856	1363.7936	0.0921
VAVLQALASTVHR	75.53	683.4087	1364.8028	1363.7936	1.0093
VAVLQALASTVHR	71.42	683.6577	1365.3008	1363.7936	1.5073
AGHQLGVTWR	48.37	562.9377	1123.8608	1123.5887	0.2722
AHTQALSMYTELLNNR	142.62	931.8597	1861.7048	1860.9152	0.7897
AHTQALSMYTELLNNR	46.42	621.7182	1862.1328	1860.9152	1.2176
AHTQALSMYTELLNNR	112.05	932.2712	1862.5278	1860.9152	1.6127
AHTQALSMYTELLNNR	105.56	932.7507	1863.4868	1860.9152	2.5717
AHTQALSMYTELLNNR	121.81	939.7942	1877.5738	1876.9101	0.6637
ADVHTFNSLIEATALVVNAK	132.12	1057.146	2112.2774	2112.1215	0.1559
ADVHTFNSLIEATALVVNAK	113.86	1057.845	2113.6754	2112.1215	1.5539
WNNILDLLK	51.08	565.1191	1128.2236	1127.6339	0.5898

Peptide Seq.	Score	m/z	Exp. Mr	Calc. Mr	Pep. Δ
**PTCD3 peptides in 55S cont'd.**					
WNNILDLLK	53.05	565.3401	1128.6656	1127.6339	1.0318
WNNILDLLK	53.44	565.3582	1128.7018	1127.6339	1.068
GSSLIIYDIMDEITGK	103.69	886.2881	1770.5616	1769.8757	0.6859
GSSLIIYDIMDEITGK	100.86	886.3376	1770.6606	1769.8757	0.7849
DPDDDMFFQSAMR	106.08	788.2272	1574.4398	1573.6177	0.8221
DPDDDMFFQSAMR	83.1	796.1401	1590.2656	1589.6127	0.653
DPDDDMFFQSAMR	92.7	796.2352	1590.4558	1589.6127	0.8432
DPDDDMFFQSAMR	95.77	803.7817	1605.5488	1605.6076	-0.0587
DLELAYQVHGLLNTGDNR	113.97	1014.899	2027.7834	2027.0072	0.7762
DLELAYQVHGLLNTGDNR	93.95	676.9832	2027.9278	2027.0072	0.9206
DLELAYQVHGLLNTGDNRK	101.27	1078.908	2155.8014	2155.1022	0.6993
DLELAYQVHGLLNTGDNRK	70.66	719.7159	2156.1259	2155.1022	1.0237
LEMIPQIWK	46.47	579.3896	1156.7646	1156.6314	0.1332
LEMIPQIWK	46.69	587.0917	1172.1688	1172.6263	-0.4575
SDLKEEILMLMAR	58.11	791.2646	1580.5146	1579.7949	0.7197
SDLKEEILMLMAR	81.55	775.3467	1548.6788	1547.8051	0.8738
SDLKEEILMLMAR	74.61	783.2892	1564.5638	1563.8	0.7638
SDLKEEILMLMAR	70.11	783.3246	1564.6346	1563.8	0.8346
SDLKEEILMLMAR	46.84	522.8365	1565.4877	1563.8	1.6877
EEILMLMAR	56.73	553.6042	1105.1938	1104.5671	0.6268
NELLNEFMDSAK	68.29	706.2687	1410.5228	1409.6496	0.8732
NELLNEFMDSAK	50.35	706.2722	1410.5298	1409.6496	0.8802
NELLNEFMDSAK	80.47	706.2762	1410.5378	1409.6496	0.8882
NELLNEFMDSAK	53.1	706.6871	1411.3596	1409.6496	1.71
NELLNEFMDSAK	72.62	706.8121	1411.6096	1409.6496	1.96
NELLNEFMDSAK	68.6	714.2247	1426.4348	1425.6446	0.7903
NELLNEFMDSAK	61.02	714.6472	1427.2798	1425.6446	1.6353
ASSSPAQAVEVVK	76.43	636.7187	1271.4228	1271.6721	-0.2493
ASSSPAQAVEVVK	73.26	637.1982	1272.3818	1271.6721	0.7097
ASSSPAQAVEVVK	74.99	637.3337	1272.6528	1271.6721	0.9807
ASSSPAQAVEVVK	47.17	638.2047	1274.3948	1271.6721	2.7227
LTADFTLSQEQK	55.52	691.1761	1380.3376	1379.6933	0.6444
LTADFTLSQEQK	83.88	691.2202	1380.4258	1379.6933	0.7326
**ICT1 Peptides in 28S**					
QGNDDIPVDR	78.35	564.8397	1127.6648	1127.5207	0.1441
QGNDDIPVDR	86.69	565.2792	1128.5438	1127.5207	1.0231
QGNDDIPVDR	83.76	565.7927	1129.5708	1127.5207	2.0501
QGNDDIPVDR	46.35	564.2717	1126.5288	1127.5207	-0.9919
QGNDDIPVDR	45.57	565.8152	1129.6158	1127.5207	2.0951
AGELILTSEYSR	73.73	670.2261	1338.4376	1337.6827	0.755
AGELILTSEYSR	74.73	670.1161	1338.2176	1337.6827	0.535
AGELILTSEYSR	67.67	669.9631	1337.9116	1337.6827	0.229
AGELILTSEYSR	67.65	670.2277	1338.4408	1337.6827	0.7582
AGELILTSEYSR	69.33	670.3057	1338.5968	1337.6827	0.9142
AGELILTSEYSR	56.81	671.0746	1340.1346	1337.6827	2.452
AGELILTSEYSR	75.05	670.3422	1338.6698	1337.6827	0.9872

Peptide Seq.	Score	m/z	Exp. Mr	Calc. Mr	Pep. Δ
**ICT1 peptides in 28S cont'd.**					
GADTAWRVPGDAK	48.58	673.0436	1344.0726	1342.663	1.4097
FHLASADWIAEPVR	118.02	806.8347	1611.6548	1610.8205	0.8343
FHLASADWIAEPVR	116.35	806.7827	1611.5508	1610.8205	0.7303
VPGDAKQGNDDIPVDR	55.53	565.2789	1692.8149	1694.8224	-2.0075
VPGDAKQGNDDIPVDR	52.31	565.7925	1694.3557	1694.8224	-0.4667
SAYSLDKLYPESR	66.82	765.1627	1528.3108	1527.7569	0.554
SAYSLDKLYPESR	71.37	765.2101	1528.4056	1527.7569	0.6488
IRDMIAEASQPATEPSKEDAALQK	111.9	873.0742	2616.2008	2614.2908	1.91
DMIAEASQPATEPSK	116.23	788.2112	1574.4078	1573.7294	0.6785
DMIAEASQPATEPSKEDAALQK	69.15	778.1415	2331.4027	2329.1107	2.292
DMIAEASQPATEPSKEDAALQK	51.56	783.4202	2347.2388	2345.1056	2.1332
**ICT1 peptides in 39S**					
QGNDDIPVDR	81.23	565.1252	1128.2358	1127.5207	0.7151
QGNDDIPVDR	49.41	565.5197	1129.0248	1127.5207	1.5041
QGNDDIPVDR	73.51	565.2097	1128.4048	1127.5207	0.8841
QGNDDIPVDR	64.93	565.5237	1129.0328	1127.5207	1.5121
QGNDDIPVDR	83.83	566.1647	1130.3148	1127.5207	2.7941
AGELILTSEYSR	84.31	670.2037	1338.3928	1337.6827	0.7102
AGELILTSEYSR	83.47	670.2967	1338.5788	1337.6827	0.8962
AGELILTSEYSR	70.23	670.1682	1338.3218	1337.6827	0.6392
AGELILTSEYSR	71.52	670.3066	1338.5986	1337.6827	0.916
AGELILTSEYSR	87.89	670.0652	1338.1158	1337.6827	0.4332
AGELILTSEYSR	73.86	669.9687	1337.9228	1337.6827	0.2402
AGELILTSEYSR	79.32	670.3876	1338.7606	1337.6827	1.078
AGELILTSEYSR	66.83	670.8367	1339.6588	1337.6827	1.9762
GADTAWRVPGDAK	58.21	672.6631	1343.3116	1342.663	0.6487
GADTAWRVPGDAK	57.69	672.5892	1343.1638	1342.663	0.5009
FHLASADWIAEPVR	108.04	806.8577	1611.7008	1610.8205	0.8803
FHLASADWIAEPVR	114.96	806.7822	1611.5498	1610.8205	0.7293
SAYSLDKLYPESR	71.03	765.2682	1528.5218	1527.7569	0.765
SAYSLDKLYPESR	45.4	510.6248	1528.8526	1527.7569	1.0957
SAYSLDKLYPESR	77.43	766.2662	1530.5178	1527.7569	2.761
VPGDAKQGNDDIPVDR	69.51	565.1252	1692.3538	1694.8224	-2.4686
VPGDAKQGNDDIPVDR	58.55	565.2098	1692.6076	1694.8224	-2.2148
VPGDAKQGNDDIPVDR	54.35	565.5239	1693.5499	1694.8224	-1.2725
VPGDAKQGNDDIPVDR	50.16	566.1649	1695.4729	1694.8224	0.6505
SAYSLDKLYPESR	85.59	765.3276	1528.6406	1527.7569	0.8838
SAYSLDKLYPESR	85.42	766.3326	1530.6506	1527.7569	2.8938
**ICT1 peptides in 55S**					
QGNDDIPVDR	61.74	565.1921	1128.3696	1127.5207	0.8489
QGNDDIPVDR	83.91	564.9241	1127.8336	1127.5207	0.3129
QGNDDIPVDR	76.46	565.6426	1129.2706	1127.5207	1.7499
QGNDDIPVDR	83.83	565.6462	1129.2778	1127.5207	1.7571
QGNDDIPVDR	83.8	565.2676	1128.5206	1127.5207	0.9999
AGELILTSEYSR	76	670.2347	1338.4548	1337.6827	0.7722

Peptide Seq.	Score	m/z	Exp. Mr	Calc. Mr	Pep. Δ
**ICT1 peptides in 55S cont'd.**					
AGELILTSEYSR	70.2	670.0992	1338.1838	1337.6827	0.5012
AGELILTSEYSR	75.82	670.1266	1338.2386	1337.6827	0.556
AGELILTSEYSR	78.53	670.3162	1338.6178	1337.6827	0.9352
AGELILTSEYSR	73.6	670.1807	1338.3468	1337.6827	0.6642
AGELILTSEYSR	75.84	670.0016	1337.9886	1337.6827	0.306
AGELILTSEYSR	70.2	670.0992	1338.1838	1337.6827	0.5012
GADTAWRVPGDAK	51.07	672.6791	1343.3436	1342.663	0.6807
FHLASADWIAEPVR	105.45	806.8226	1611.6306	1610.8205	0.8101
VPGDAKQGNDDIPVDR	65.56	565.6425	1693.9057	1694.8224	-0.9167
VPGDAKQGNDDIPVDR	55.61	565.6458	1693.9156	1694.8224	-0.9068
VPGDAKQGNDDIPVDR	55.22	565.2675	1692.7807	1694.8224	-2.0417
SAYSLDKLYPESR	67.94	765.3112	1528.6078	1527.7569	0.851
SAYSLDKLYPESR	78.97	765.2617	1528.5088	1527.7569	0.752
SAYSLDKLYPESR	64.16	766.2731	1530.5316	1527.7569	2.7748
DMIAEASQPATEPSKEDAALQK	122.14	1165.934	2329.8534	2329.1107	0.7427
DMIAEASQPATEPSKEDAALQK	67.03	777.7215	2330.1427	2329.1107	1.032
**CRIF1 peptides in 28S**					
HGAASGVDPGSLWPSR	74.51	797.7452	1593.4758	1592.7696	0.7063
AAAMAAAAAQDPADSETPDS	109.97	939.0322	1876.0498	1875.7792	0.2706
AAAMAAAAAQDPADSETPDS	90.74	626.6055	1876.7947	1875.7792	1.0155
AAAMAAAAAQDPADSETPDS	108.94	939.3212	1876.6278	1875.7792	0.8486
AAAMAAAAAQDPADSETPDS	99.52	627.1435	1878.4087	1875.7792	2.6295
MPQMIENWR	45.54	619.7411	1237.4676	1235.5427	1.925
FQELLQDLEK	61.06	631.5262	1261.0378	1261.6554	-0.6175
FQELLQDLEK	64.88	632.1782	1262.3418	1261.6554	0.6865
FQELLQDLEK	46.01	632.2496	1262.4846	1261.6554	0.8293
FQELLQDLEK	61.91	632.3481	1262.6816	1261.6554	1.0263
FQELLQDLEK	64.96	631.7542	1261.4938	1261.6554	-0.1615
FQELLQDLEK	68.55	632.7212	1263.4278	1261.6554	1.7725
FQELLQDLEKQHR	85.2	842.7342	1683.4538	1682.874	0.5799
**CRIF1 peptides in 39S**					
HGAASGVDPGSLWPSR	66.06	532.4785	1594.4137	1592.7696	1.6441
HGAASGVDPGSLWPSR	86.51	798.2892	1594.5638	1592.7696	1.7943
HGAASGVDPGSLWPSR	74.96	532.6932	1595.0578	1592.7696	2.2882
HGAASGVDPGSLWPSR	85.67	797.7416	1593.4686	1592.7696	0.6991
HGAASGVDPGSLWPSR	96.4	797.7676	1593.5206	1592.7696	0.7511
HGAASGVDPGSLWPSR	71.57	532.4385	1594.2937	1592.7696	1.5241
HGAASGVDPGSLWPSR	78.66	532.9279	1595.7619	1592.7696	2.9923
HGAASGVDPGSLWPSR	97.01	797.7322	1593.4498	1592.7696	0.6803
HGAASGVDPGSLWPSR	72.81	532.3282	1593.9628	1592.7696	1.1932
HGAASGVDPGSLWPSR	70.92	532.3935	1594.1587	1592.7696	1.3891
HGAASGVDPGSLWPSR	84.54	798.3267	1594.6388	1592.7696	1.8693
HGAASGVDPGSLWPSR	52.4	798.7756	1595.5366	1592.7696	2.7671
AAAMAAAAAQDPADSETPDS	114.83	931.1351	1860.2556	1859.7843	0.4713

Peptide Seq.	Score	m/z	Exp. Mr	Calc. Mr	Pep. Δ
**CRIF1 peptides in 39S cont'd.**					
AAAMAAAAAQDPADSETPDS	103.72	939.4617	1876.9088	1875.7792	1.1296
MPQMIENWR	45.93	611.2447	1220.4748	1219.5478	0.9271
MPQMIENWR	46.69	611.7496	1221.4846	1219.5478	1.9369
MPQMIENWR	48.96	603.0107	1204.0068	1203.5529	0.454
MPQMIENWR	48.27	619.7856	1237.5566	1235.5427	2.014
MPQMIENWR	55.05	619.1967	1236.3788	1235.5427	0.8362
MPQMIENWR	50.42	619.7457	1237.4768	1235.5427	1.9342
MPQMIENWR	49.69	603.3232	1204.6318	1203.5529	1.079
FQELLQDLEK	53.53	1262.775	1261.7677	1261.6554	0.1123
FQELLQDLEK	60.84	631.9736	1261.9326	1261.6554	0.2773
FQELLQDLEK	60.13	632.2322	1262.4498	1261.6554	0.7945
FQELLQDLEK	64.76	632.3097	1262.6048	1261.6554	0.9495
EQLLELEAEER	58.31	680.2726	1358.5306	1357.6725	0.8582
EQLLELEAEER	65.32	679.9182	1357.8218	1357.6725	0.1494
EQLLELEAEER	62.22	680.2352	1358.4558	1357.6725	0.7834
EQLLELEAEER	62.32	680.2886	1358.5626	1357.6725	0.8902
EQLLELEAEER	74.92	679.9457	1357.8768	1357.6725	0.2044
FQELLQDLEK	65	632.0447	1262.0748	1261.6554	0.4195
FQELLQDLEK	64.9	631.8091	1261.6036	1261.6554	-0.0517
FQELLQDLEK	64.78	632.3376	1262.6606	1261.6554	1.0053
FQELLQDLEK	61.75	632.2822	1262.5498	1261.6554	0.8945
FQELLQDLEK	56.81	632.2986	1262.5826	1261.6554	0.9273
FQELLQDLEK	68.34	632.5237	1263.0328	1261.6554	1.3775
**CRIF1 peptides in 55S**					
HGAASGVDPGSLWPSR	97.4	797.4601	1592.9056	1592.7696	0.1361
HGAASGVDPGSLWPSR	69.43	532.2872	1593.8398	1592.7696	1.0702
HGAASGVDPGSLWPSR	68.34	532.3582	1594.0528	1592.7696	1.2832
HGAASGVDPGSLWPSR	85.09	798.2792	1594.5438	1592.7696	1.7743
HGAASGVDPGSLWPSR	111.3	797.5356	1593.0566	1592.7696	0.2871
HGAASGVDPGSLWPSR	71.97	532.0828	1593.2266	1592.7696	0.457
HGAASGVDPGSLWPSR	63.57	532.5385	1594.5937	1592.7696	1.8241
HGAASGVDPGSLWPSR	56.35	532.8549	1595.5429	1592.7696	2.7733
HGAASGVDPGSLWPSR	80.56	532.0665	1593.1777	1592.7696	0.4081
HGAASGVDPGSLWPSR	80.07	532.1508	1593.4306	1592.7696	0.661
HGAASGVDPGSLWPSR	92.12	797.7886	1593.5626	1592.7696	0.7931
HGAASGVDPGSLWPSR	95.3	797.8016	1593.5886	1592.7696	0.8191
HGAASGVDPGSLWPSR	83.18	532.7745	1595.3017	1592.7696	2.5321
HGAASGVDPGSLWPSR	70.83	532.8608	1595.5606	1592.7696	2.791
AAAMAAAAAQDPADSETPDS	115.6	930.9097	1859.8048	1859.7843	0.0205
AAAMAAAAAQDPADSETPDS	105.18	932.1652	1862.3158	1859.7843	2.5315
AAAMAAAAAQDPADSETPDS	121.4	939.1917	1876.3688	1875.7792	0.5896
AAAMAAAAAQDPADSETPDS	72.23	939.8972	1877.7798	1875.7792	2.0006
EQLLELEAEER	63.32	679.9067	1357.7988	1357.6725	0.1264
EQLLELEAEER	65.43	680.2437	1358.4728	1357.6725	0.8004

Peptide Seq.	Score	m/z	Exp. Mr	Calc. Mr	Pep. Δ
**CRIF1 peptides in 55S cont'd.**					
EQLLELEAEER	73.71	680.2897	1358.5648	1357.6725	0.8924
MPQMIENWR	46.05	602.9772	1203.9398	1203.5529	0.387
MPQMIENWR	46.49	610.4427	1218.8708	1219.5478	-0.6769
MPQMIENWR	46.25	619.2236	1236.4326	1235.5427	0.89
MPQMIENWR	53.57	620.2697	1238.5248	1235.5427	2.9822
MPQMIENWR	54.25	602.8962	1203.7778	1203.5529	0.225
MPQMIENWR	54.9	619.5807	1237.1468	1235.5427	1.6042
MPQMIENWR	54.94	620.0892	1238.1638	1235.5427	2.6212
MPQMIENWR	52.36	602.8542	1203.6938	1203.5529	0.141
MPQMIENWR	55.34	618.9382	1235.8618	1235.5427	0.3192
MPQMIENWR	54.96	620.1552	1238.2958	1235.5427	2.7532
FQELLQDLEK	55.59	631.7546	1261.4946	1261.6554	-0.1607
FQELLQDLEK	65.12	631.9077	1261.8008	1261.6554	0.1455
FQELLQDLEK	64.85	632.7242	1263.4338	1261.6554	1.7785
FQELLQDLEK	56.47	632.3876	1262.7606	1261.6554	1.1053
FQELLQDLEK	55.77	631.9131	1261.8116	1261.6554	0.1563
FQELLQDLEK	60.12	632.1141	1262.2136	1261.6554	0.5583

M: Methionine oxidation
